# Cancer-related mortality among individuals with schizophrenia: systematic review and meta-analysis of cohort studies

**DOI:** 10.1192/bjo.2026.12068

**Published:** 2026-08-06

**Authors:** Seyyed Muhammad Mahdi Mahdavinoor, Aghil Mollaei, Arash Golzar, Saeed Kargar-Soleimanabad, Amir-Hassan Bordbari, Melika Amiri, Mohammad Hosein Rafiee, Leila Seddigh

**Affiliations:** Faculty of Allied Medical Sciences, Mazandaran University of Medical Sciences, Sari, Iran; Student Research Committee, Faculty of Health, Mazandaran University of Medical Sciences, Sari, Iran; Department of Clinical Psychology, Faculty of Medicine, Islamic Azad University of Qom, Qom, Iran; Student Research Committee, Faculty of Medicine, Mazandaran University of Medical Sciences, Sari, Iran; Student Research Committee, Department of Psychology, Tehran Medical Branch, Islamic Azad University, Tehran, Iran; Faculty of Nursing and Midwifery, Tabriz University of Medical Sciences, Tabriz, Iran; Department of Community Medicine, Tehran University of Medical Sciences, Tehran, Iran

**Keywords:** Schizophrenia, cancer, mortality, meta-analysis, neoplasms

## Abstract

**Background:**

Individuals with schizophrenia have shown distinct cancer incidence patterns.

**Aims:**

We aimed to assess whether their cancer-related mortality differs from the general population overall, by gender and by specific cancer types.

**Method:**

We systematically searched Scopus, Web of Science, PsycINFO, PubMed and Embase, up to December 2025. Each record was independently screened by two reviewers, and data were independently extracted by two investigators. Two authors assessed the quality of the included articles with the Newcastle–Ottawa Scale. Analyses were conducted using Stata version 16; between-study heterogeneity was evaluated with Cochran’s *Q*-statistic and the *I* ^2^-statistic, and potential sources of heterogeneity were further examined through exploratory meta-regression.

**Results:**

Meta-analysis of cohort studies showed that individuals with schizophrenia had a 55% higher risk of cancer-related death than the general population (standardised mortality ratio (SMR) 1.55; 95% CI 1.16–2.07). In gender-stratified analyses, SMRs for all cancers combined were 1.37 (95% CI 1.01–1.87) in men and 1.43 (95% CI 1.15–1.79) in women. Site-specific SMRs were 1.77 (95% CI 1.17–2.68) for breast, 2.40 (95% CI 2.35–2.45) for respiratory, 1.54 (95% CI 1.35–1.76) for gastrointestinal, 2.32 (95% CI 0.72–7.45) for haematologic, 4.43 (95% CI 0.71–27.62) for skin and soft tissue, 3.03 (95% CI 1.96–4.69) for urogenital and 1.01 (95% CI: 0.36–2.83) for other cancers, although precision varied considerably across cancer sites.

**Conclusions:**

Schizophrenia was associated with substantially elevated cancer-related mortality compared with the general population. These findings underscore the need for earlier cancer detection and guideline-concordant, integrated physical and mental healthcare for this high-risk group.

Schizophrenia is one of the most severe psychiatric disorders. It is chronic and complex, and occurs across all societies and socioeconomic strata.^1,2^ The raw estimates of prevalence, incidence and burden of schizophrenia have been increasing since 1990. From 1990 to 2019, crude prevalence (from 14.2 million to 23.6 million), incidence cases (from 941 000 to 1.3 million), and disability-adjusted life-years (from 9.1 million to 15.1 million) rose by more than 65, 37 and 65%, respectively.^3^ Given the large number of people affected by schizophrenia, the difficulties it creates for patients and their families, and the substantial costs it imposes on society, schizophrenia is considered a major public health concern rather than merely a disorder.^4–7^ Individuals with schizophrenia have a substantially shorter life expectancy than the general population (14.5 years of potential life lost; 95% CI 11.2–17.8).^8^ Multiple factors contribute to reduced life expectancy in this population, including unhealthy and sedentary lifestyles,^9^ delayed diagnosis and discontinuous treatment,^10,11^ lower cognitive functioning,^12^ increased risk of certain cancers,^13^ diabetes,^14^ Parkinson’s disease^15^ and other conditions.

In recent decades, the association between schizophrenia and cancer has garnered increasing attention. Although some studies suggest that individuals with schizophrenia have a lower risk of developing certain cancers – such as liver,^16^ prostate^17,18^ and colorectal cancer^19^ – than the general population, other studies indicate higher susceptibility to others, such as breast cancer.^13^ For some cancers (e.g. lung cancer), the incidence does not differ significantly from that in the general population.^20^ The relationship between schizophrenia and cancer risk appears complex, and is likely shaped by both genetic and environmental factors. For example, one specific gene has been proposed as a possible contributor to the lower risk of colorectal cancer observed in individuals with schizophrenia.^21^ In addition, people with schizophrenia often have unhealthy and sedentary lifestyles, which can lead to various disorders and may influence cancer risk and outcomes.^9,13^ Given this complex interplay, together with condition-specific challenges such as impaired reality testing, discontinuous treatment and delayed diagnosis, precise estimates of cancer-related mortality among individuals with schizophrenia remain uncertain.

Prior studies have addressed this question, but primary studies reported conflicting results. For example, Ko and colleagues^22^ found cancer-related mortality to be markedly higher among individuals with schizophrenia than in the general population (standardised mortality ratio (SMR) 4.8, 95% CI 3.5–5.9), whereas Perini and colleagues^23^ reported the opposite pattern (SMR 0.83, 95% CI 0.5–1.3).

Arguably, the most important study to date is by Zhuo and colleagues, who synthesised cohort studies published through mid-2016 and concluded that cancer-related mortality was significantly higher in individuals with schizophrenia than in the general population (SMR 1.40, 95% CI 1.29–1.52, *P* < 0.001).^24^ Despite its value, that meta-analysis has limitations that preclude relying on its estimates at present. First, the search covered only PubMed and Embase, so eligible studies may have been missed; in addition, new studies have been published since then, and several cohorts may have been updated. Second, the authors did not assess the quality or risk of bias of the included studies, leaving uncertainty about the credibility of the pooled evidence. Third, despite substantial heterogeneity, potential sources of heterogeneity were not identified. Moreover, given that associations between schizophrenia and specific cancer sites are not uniform, reporting site-specific cancer mortality would have been informative, but was not undertaken. In light of these gaps and the passage of time, we designed a new study to estimate cancer-related mortality among individuals with schizophrenia.

## Method

### Review question

Our review aimed to answer the question of whether individuals with schizophrenia have a higher risk of cancer-related mortality compared with the general population, both overall and across specific cancer sites.

### Study design

We developed and conducted this systematic review and meta-analysis in accordance with the Cochrane Handbook for Systematic Reviews of Interventions,^25^ and a step-by-step guide for conducting a systematic review and meta-analysis with simulation data.^26^ We also reported this study in accordance with the Preferred Reporting Items for Systematic Reviews and Meta-Analyses (PRISMA) guidelines,^27^ and additionally considered the Reporting Guidelines for Meta-Analyses of Observational Studies (MOOSE) guidelines.^28^ The protocol was preregistered with the International Registration of Systematic Reviews (PROSPERO) under the following registration number: CRD42024613495.

### Search strategy to identify relevant studies

We conducted both electronic and manual searches, performing a comprehensive systematic search across international databases, including Web of Science, PubMed, Scopus, Embase and PsycINFO, up to 12 November 2024. After that, we updated the search until 10 December 2025. The search strategy incorporated relevant keywords and MeSH terms: [(Mortality OR Death) AND (Cancer* OR Neoplasm* OR Tumor* OR Malignancy OR Carcinoma* OR Gland) AND (‘Dementia Praecox’ OR ‘Schizophre*’ OR ‘psychosis’)]. No restrictions were applied regarding the publication year or region, but only studies published in English were included. Additionally, we manually reviewed the reference lists of the included studies to identify any potentially relevant research that might have been missed in the database search.

### Risk-of-bias assessment

Two authors (A.-H.B. and S.M.M.M.) independently evaluated the quality of each included study, using the Newcastle–Ottawa Scale (NOS).^29^ This scale awards up to nine stars, with higher scores indicating better quality, and evaluates studies based on group selection, comparability of groups and outcome determination. Studies with a score of ≥7 were classified as good quality, those with a score of 6 were classed as fair quality and those with a score of <6 were classed as poor quality. Any discrepancies were resolved through consensus.

### Inclusion and exclusion criteria

The included studies met the following criteria: (a) cohort studies, (b) full-text articles published in peer-reviewed English journals, (c) study populations consisting of individuals diagnosed with schizophrenia at baseline, (d) studies that included a control group from the general population without a schizophrenia diagnosis and (e) studies that reported cancer-related mortality in patients with schizophrenia. Studies were excluded if they met any of the following criteria: (a) any article type other than cohort studies and (b) duplicate publications.

In cases of overlapping studies, the one with the longest follow-up duration and largest sample size was selected.

### Screening and data extraction

Each record was independently screened and selected by two reviewers (A.G., S.K.-S. and M.A.). Data were independently extracted by two other investigators (L.S. and M.H.R.). At each stage, disagreements were resolved through consultation with another author who was not involved in the initial assessment (S.M.M.M.). We extracted the following data: first author, year of publication, country, number of individuals with schizophrenia, method of schizophrenia ascertainment, comparison population, follow-up duration, number of cancer deaths among individuals with schizophrenia, method of ascertainment of cancer deaths, cancer type (site) and effect measure (SMR, mortality rate ratio (MRR), risk ratio).

### Strategy for data synthesis

We summarised cancer-related mortality by using SMRs with 95% confidence intervals for each cancer type in individuals with schizophrenia. For meta-analysis, SMRs and their 95% confidence intervals were transformed to the natural logarithmic scale. When 95% confidence intervals were reported, standard errors were calculated from the log-transformed confidence limits by using the formula s.e. = (natural logarithm upper confidence interval − natural logarithm lower confidence interval) / (2 × 1.96). When 95% confidence intervals were not reported but sufficient information was available, confidence intervals were estimated with the approach described by Breslow and Day.^30^ Pooled estimates were then back-transformed to the original scale using the exponential function. Because a sufficient number of studies reported SMRs, we did not include studies that presented only risk ratios or MRRs in the quantitative meta-analysis, to maintain consistency and precision in the effect measure. However, studies reporting risk ratios or MRRs were retained in the systematic review and data extraction table. Pooled estimates were obtained with random-effects meta-analysis. Between-study heterogeneity was assessed with Cochran’s *Q*-statistic and quantified with the *I* ^2^-statistic. When heterogeneity was substantial (*I* ^2^ > 50%), we conducted exploratory meta-regression analyses to investigate potential sources of heterogeneity. Meta-regression was performed only for analyses that included five or more studies. This threshold was used as a pragmatic decision to avoid conducting meta-regression in very sparse analyses. However, these analyses were still considered exploratory because statistical power may remain limited even when five or more studies are available. Potential publication bias was evaluated with Begg’s rank correlation and Egger’s regression tests in analyses including more than two studies, together with visual inspection of funnel plots. All analyses were performed using Stata/MP version 16.0 for Windows (StataCorp LLC, College Station, Texas, USA).

## Results

### Literature search

**Fig. 1:**
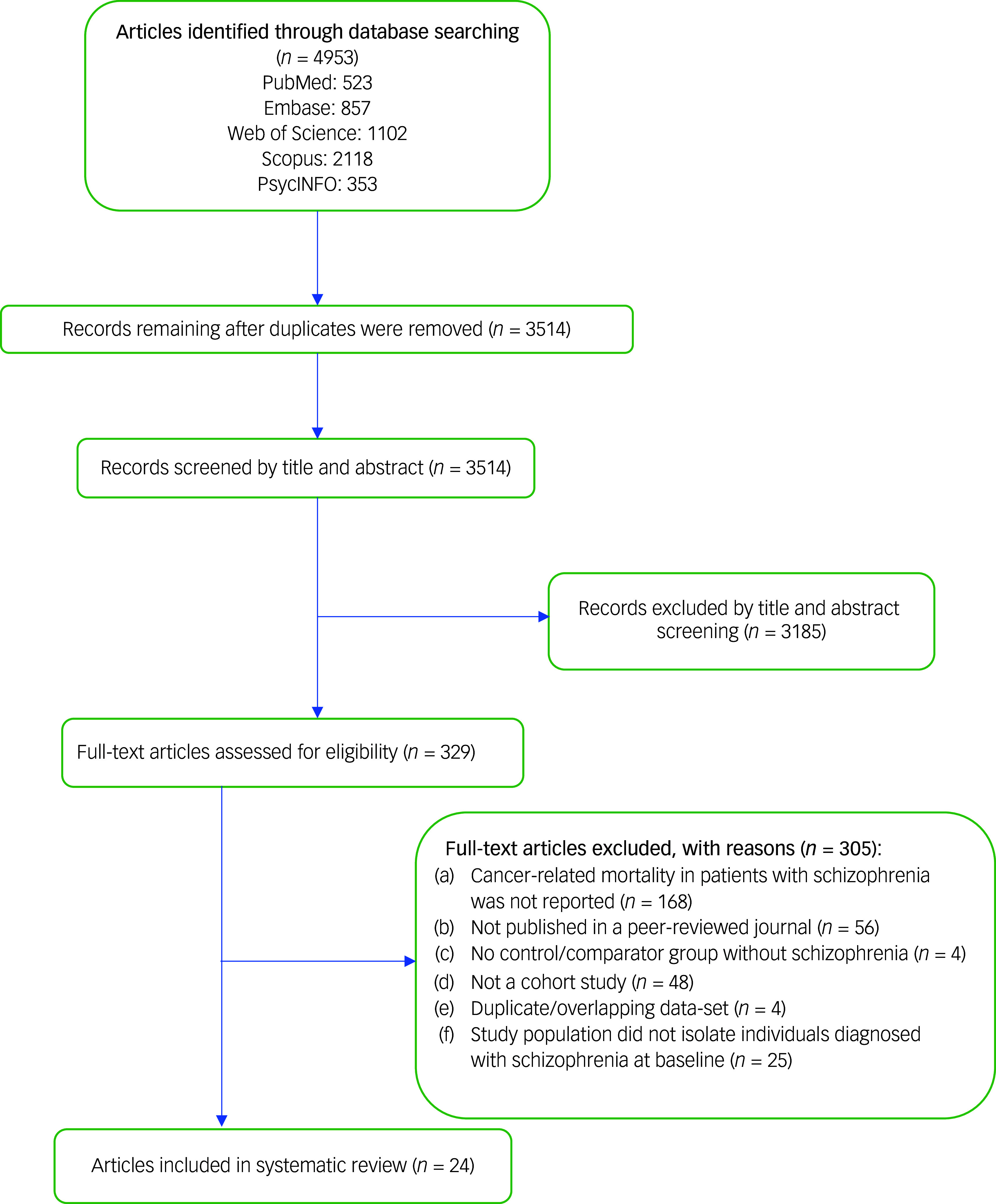
Preferred Reporting Items for Systematic Reviews and Meta-Analyses flowchart for study selection process.

After searching various databases, a total of 4953 articles were identified. Subsequently, 1439 duplicate records were removed. Two reviewers independently screened the titles and abstracts of the remaining 3514 articles, resulting in the exclusion of 3185 records because of irrelevance. The full texts of the remaining 329 articles were then assessed independently by two reviewers. Of these, 305 articles were excluded for not meeting the inclusion criteria or meeting at least one exclusion criterion. Finally, 24 articles were included in the study. The process of literature search, study inclusion and exclusion is illustrated in Fig. 1.

### Study characteristics

Finally, 24 articles^22,23,31–52^ were included in the study. The publication years of the included studies ranged from 1991 to 2025. A total of 13 studies were conducted in European countries,^23,31,34–38,41,43,45–47,50^ reflecting considerable attention to this issue in that region. In contrast, only five studies were conducted in Asia,^22,39,40,51,52^ despite the fact that this continent accounts for over 58% of the world’s population.^53^ Also, North America^33,42,48^ and Australia^32,44,49^ each contributed three studies. Most of the included studies reported SMRs, one study reported MRRs^46^ and six studies reported risk ratios.^32,33,37,44,45,49^ Only studies reporting SMRs were included in the pooled meta-analyses; studies reporting only MRRs or risk ratios were retained in the systematic review and presented in the data extraction table. Overall, 24 articles provided effect size estimates for cancer-related mortality in individuals with schizophrenia. Additionally, seven studies reported effect sizes for specific cancer types. The included studies reported a wide range of cancer sites. Therefore, we grouped specific cancer sites/types into broader categories to make the results more clinically interpretable and easier to synthesise. Several alternative classifications are possible. However, our grouping was based on the cancer types reported in the included studies, the amount of available data for each site and clinical or anatomical similarity across cancer sites. Some included studies also reported categories such as ‘other’, ‘unspecified’ or ‘ill-defined’ cancers. These categories were retained and grouped under ‘other/ill-defined cancers’. Accordingly, cancer types were categorised into eight groups: breast, central nervous system, gastrointestinal, haematologic, respiratory, skin and soft tissue, urogenital and other/ill-defined cancers. The data extraction is shown in Supplementary Table 1 available online at https://doi.org/10.1192/bjo.2026.12068.

### Methodological quality

Among the included articles, 22 were classified as being of good quality and two were classified as being of fair quality. Full details of the quality assessment for each article can be found in the Supplementary Material.

### Overall and site-specific cancer-related mortality in patients with schizophrenia

Random-effects meta-analyses showed that, across all cancer types examined, individuals with schizophrenia had higher cancer-related mortality than the general population. However, this increase was not statistically significant for all cancer sites (gastrointestinal cancer in men: *P* = 0.98, SMR 1.01, 95% CI 0.62–1.63; haematological cancers in both genders: *P* = 0.16, SMR 2.32, 95% CI 0.72–7.45; skin and soft tissue cancers in both genders: *P* = 0.11, SMR 4.43, 95% CI 0.71–27.62; skin and soft tissue cancers in men: *P* = 0.88, SMR 1.12, 95% CI 0.27–4.57; skin and soft tissue cancers in women: *P* = 0.79, SMR 1.08, 95% CI 0.60–1.97; urogenital cancer in men: *P* = 0.09, SMR 1.73, 95% CI 0.93–3.23; other cancers in both genders: *P* = 0.99, SMR 1.01, 95% CI 0.36–2.83). Meta-analysis of studies that reported overall cancer-related mortality across all cancer types indicated that individuals with schizophrenia had a 55% higher risk of cancer-related death than those without schizophrenia (SMR 1.55, 95% CI 1.16–2.07). In gender-stratified analyses, cancer-related mortality was 37% higher in men with schizophrenia (SMR 1.37, 95% CI 1.01–1.87) and 43% higher in women with schizophrenia (SMR 1.43, 95% CI 1.15–1.79) compared with the corresponding general population. Detailed random-effects meta-analytic estimates by cancer site are presented in Table 1, and the corresponding forest plots are shown in Supplementary Figs 1–23.

**Table 1:** Pooled standardised mortality ratios for cancer-related mortality in individuals with schizophrenia, by cancer type and gender (summary of effect sizes from forest plots; corresponding forest plots are presented in the Supplementary Figs) Table 1 long description.

Cancer type	Number of studies	Pooled SMR	95% CI	*P*-value	*I*^2^ %	*P*-value
All	10	1.55	1.16–2.07	<0.001	98.16	<0.001
Male	12	1.37	1.01–1.87	<0.001	97.18	<0.001
Female	13	1.43	1.15–1.79	<0.001	95.50	<0.001
Breast	4	1.77	1.17–2.68	0.001	45.63	0.070
Male	1	3.60	1.8–5.4	<0.001	–	–
Female	4	1.72	1.48–1.99	<0.001	32.20	0.170
Respiratory	5	2.40	2.35–2.45	<0.001	0.00	0.110
Male	6	1.42	0.72–2.81	<0.001	95.60	<0.001
Female	2	1.94	1.07–3.54	0.030	80.33	0.020
Gastrointestinal	11	1.54	1.35–1.76	<0.001	52.20	0.010
Male	17	1.01	0.62–1.63	0.980	95.51	<0.001
Female	16	1.55	1.15–2.07	<0.001	82.19	<0.001
Haematologic	4	2.32	0.72–7.45	0.160	67.63	0.030
Male	7	1.39	1.26–1.54	<0.001	0.00	0.920
Female	5	1.50	1.32–1.71	<0.001	0.00	0.240
Other	2	1.01	0.36–2.83	0.990	72.60	0.060
Male	3	1.41	1.23–1.60	<0.001	0.00	0.220
Female	3	1.82	1.21–2.75	<0.001	48.02	0.160
Skin and soft tissue	2	4.43	0.71–27.62	0.110	90.45	<0.001
Male	2	1.12	0.27–4.57	0.880	87.00	0.010
Female	3	1.08	0.60–1.97	0.790	7.22	0.380
Urogenital	7	3.03	1.96–4.69	<0.001	72.42	<0.001
Male	9	1.73	0.93–3.23	0.090	97.86	<0.001
Female	11	1.49	1.21–1.84	<0.001	0.00	0.520

SMR, standardised mortality ratio.

### Publication bias

To assess publication bias, we used Begg’s and Egger’s tests (Table 2), as well as funnel plots (Supplementary Figs 24–46). The quantitative tests, including Begg’s test and Egger’s test, were not significant in most of the analyses (*P* > 0.05), but this absence of statistical evidence should not be interpreted as evidence that publication bias was absent. Only in one case (haematological cancer in women) was Egger’s test statistically significant; however, given the small number of studies in that analysis (five studies), no firm conclusion about the presence of publication bias can be drawn. At the same time, some funnel plots suggested possible asymmetry. Overall, because funnel plots and Begg’s and Egger’s tests have limited power in analyses with a small number of studies, and in view of the inconsistency between the quantitative Begg’s and Egger’s tests and the funnel plots even in those instances where the number of studies was sufficient, no definitive conclusion can be reached regarding publication bias in this study.

**Table 2:** Summary of publication bias assessments (Begg’s and Egger’s tests) for the meta-analyses (corresponding funnel plots are presented in the Supplementary Figs) Table 2 long description.

Cancer type	*n*	Begg’s test	Egger’s test
All	10	*P* = 0.720	*P* = 0.853
Male	12	*P* = 0.373	*P* = 0.915
Female	13	*P* = 0.300	*P* = 0.420
Breast	4	*P* = 0.734	*P* = 0.324
Male	–	–	–
Female	4	*P* = 0.308	*P* = 0.720
Respiratory	5	*P* = 1.000	*P* = 0.509
Male	6	*P* = 1.000	*P* = 0.392
Female	–	–	–
Gastrointestinal	11	*P* = 0.309	*P* = 0.527
Male	17	*P* = 0.934	*P* = 0.191
Female	16	*P* = 0.065	*P* = 0.259
Haematologic	4	*P* = 1.000	*P* = 0.877
Male	7	*P* = 0.763	*P* = 0.663
Female	5	*P* = 0.462	*P* = 0.036
Other	–	–	–
Male	3	*P* = 1.000	*P* = 0.317
Female	3	*P* = 1.000	*P* = 0.628
Skin and soft tissue	–	–	–
Male	–	–	–
Female	3	*P* = 0.296	*P* = 0.171
Urogenital	7	*P* = 0.172	*P* = 0.081
Male	9	*P* = 0.175	*P* = 0.081
Female	11	*P* = 1.000	*P* = 0.611

### Meta-regression

We used meta-regression to examine potential sources of heterogeneity in cancer-related SMRs among individuals with schizophrenia. When all cancer types were combined (all cancers, both genders), none of the examined study-level variables (number of patients, year of publication or follow-up duration) was significantly associated with the SMR. In the gender-stratified analyses for all cancers, the SMR in men was positively associated with year of publication (*β* = 0.038, *P* = 0.028), whereas no significant associations were observed with the number of patients or length of follow-up. Among women, the SMR showed no significant association with any of the tested covariates, suggesting that overall, all-cancer SMRs were largely stable across study characteristics, with the exception of a modest calendar-time effect in men.

For mortality owing to respiratory cancers, year of publication showed a significant inverse association with the SMR (*β* = −0.78, *P* = 0.045), meaning that more recent studies reported lower SMRs and older studies reported higher SMRs. In respiratory cancer mortality among men, follow-up duration showed a significant association with the SMR (*β* = −0.109, *P* = 0.008), indicating that follow-up duration may be a potential source of heterogeneity in SMR estimates for respiratory cancer mortality in men. In the gender-specific analysis of gastrointestinal cancers among women, all three variables – the number of patients (*β* = −0.00000048, *P* = 0.027), year of publication (*β* = −0.104, *P* < 0.001) and follow-up duration (*β* = −0.092, *P* < 0.001) – were identified as potential sources of heterogeneity, each showing a significant inverse association with the SMR. In other words, studies with larger sample sizes, more recent publication years and longer follow-up periods tended to report lower SMRs (Table 3).

**Table 3:** Meta-regression analyses exploring potential sources of heterogeneity in cancer-related mortality Table 3 long description.

Cancer type	Coefficient (*β*)	95% CI	s.e.	*P*-value
All				
Overall (both genders)				
Number of patients	0.000000221	(−0.00000105 to 0.00000105)	0.000000650	0.734
Year of publication	0.008	(−0.074 to 0.090)	0.041	0.846
Follow-up duration	0.016	(−0.054 to 0.086)	0.035	0.657
Male				
Number of patients	0.000000458	(−0.00000047 to 0.00000139)	0.000000474	0.334
Year of publication	0.038	(0.004−0.072)	0.017	**0.028**
Follow-up duration	0.036	(−0.005 to 0.077)	0.021	0.087
Female				
Number of patients	0.000000384	(−0.000000354 to 0.00000112)	0.000000377	0.308
Year of publication	0.023	(−0.005 to 0.050)	0.014	0.115
Follow-up duration	0.027	(−0.006 to 0.061)	0.017	0.118
Respiratory				
Overall (both genders)				
Number of patients	0.00000427	(−0.000000251 to 0.00000879)	0.00000231	0.064
Year of publication	−0.78	(−1.547 to −0.018)	0.389	**0.045**
Follow-up duration	0.055	(−0.059 to 0.170)	0.058	0.347
Male				
Number of patients	−0.0000008	(−0.00000221 to 0.000000605)	0.000000717	0.264
Year of publication	0.023	(−0.043 to 0.090)	0.034	0.491
Follow-up duration	−0.109	(−0.190 to −0.028)	0.041	**0.008**
Gastrointestinal				
Overall (both genders)				
Number of patients	0.000000223	(−0.000000882 to 0.00000133)	0.000000564	0.692
Year of publication	−0.127	(−0.377 to 0.123)	0.127	0.320
Follow-up duration	−0.063	(−0.378 to 0.251)	0.160	0.692
Male				
Number of patients	−0.0000000349	(−0.000000972 to 0.000000903)	0.000000478	0.942
Year of publication	−0.082	(−0.165 to 0.001)	0.043	0.055
Follow-up duration	−0.073	(−0.146 to 0.001)	0.037	0.055
Female				
Number of patients	−0.00000048	(−0.00000090 to −0.000000055)	0.00000021	**0.027**
Year of publication	−0.104	(−0.155to −0.052)	0.026	**<0.001**
Follow-up duration	−0.092	(−0.137 to −0.046)	0.023	**<0.001**
Haematologic				
Male				
Number of patients	−0.000012	(−0.0004349 to 0.0001883)	0.000159	0.438
Year of publication	−34.922	(−123.025 to 53.181)	44.951	0.437
Follow-up duration	−0.036	(−0.130 to 0.056)	0.047	0.437
Female				
Number of patients	−0.000000026	(−0.00000112 to 0.000000107)	0.000000560	0.963
Year of publication	0.007	(−0.302 to 0.317)	0.158	0.963
Follow-up duration	0.001	(−0.055 to 0.057)	0.029	0.963
Urogenital				
Overall (both genders)				
Number of patients	−0.0000134	(−0.0003941 to 0.0003674)	0.0001943	0.945
Year of publication	0.00000449	(−0.0000688 to 0.0000778)	0.0000374	0.905
Follow-up duration	0.003	(−0.072 to 0.078)	0.038	0.945
Male				
Number of patients	−0.000206	(−0.0004466 to 0.0000346)	0.0001227	0.093
Year of publication	−0.017	(−0.090 to 0.055)	0.037	0.643
Follow-up duration	−0.058	(−0.133 to 0.017)	0.038	0.130
Female				
Number of patients	−0.0001633	(−0.0004009 to 0.0000742)	0.0001212	0.178
Year of publication	−0.017	(−0.038 to 0.003)	0.011	0.101
Follow-up duration	−0.048	(−0.103 to 0.007)	0.028	0.088

Bold indicates statistical significance.

## Discussion

Our objective in this study was to examine cancer-related mortality among individuals with schizophrenia. To our knowledge, this is the first systematic review and meta-analysis to provide an updated and comprehensive synthesis of cancer-related mortality in schizophrenia simultaneously at the overall, gender-specific and broad cancer-site-specific levels, while also incorporating quality assessment and exploratory analyses of heterogeneity. Meta-analysis of ten cohort studies that reported cancer-related mortality irrespective of gender and cancer site showed that individuals with schizophrenia had a 55% higher risk of cancer-related death than the general population (SMR 1.55, 95% CI 1.16–2.07, *I*^2^ = 98.16%). In studies that reported gender-specific mortality, this excess remained evident: for men, the risk was 37% higher (SMR 1.37, 95% CI 1.01–1.87, *I*^2^ = 97.18%), and for women it was 43% higher (SMR 1.43, 95% CI 1.15–1.79, *I*^2^ = 95.50%). The discrepancy between the overall pooled estimate and the gender-specific estimates is likely to reflect the substantial heterogeneity in the body of studies. Site-specific meta-analyses likewise indicated higher cancer-related mortality among individuals with schizophrenia than in the general population, and this difference was statistically significant for most cancer types. Restricting the quantitative synthesis to SMRs improved comparability across studies, but it may have reduced the representativeness of the pooled estimates by excluding studies that reported only risk ratios or MRRs. These studies were retained in the descriptive synthesis, and their findings were broadly concordant with the overall direction of the pooled SMR estimates, although one male-specific risk ratio estimate did not show an increased risk.

In contrast to cancer-related mortality, which is higher among individuals with schizophrenia than in the general population across different cancer types, cancer incidence in this group shows a different pattern. For example, the incidence of liver cancer,^16^ prostate cancer^17,18^ and colorectal cancer^19^ has been reported to be lower in people with schizophrenia than in the general population, whereas the incidence of breast cancer^13^ appears to be higher. Nevertheless, cancer-related mortality is elevated in almost all cancer types. Diagnosis at more advanced stages of cancer and delays in the provision of care,^54^ treatment that deviates from guideline-recommended protocols^55,56^ and possible immune-inflammatory dysregulation in schizophrenia, which may contribute to cancer progression and treatment resistance,^57,58^ are likely to be important contributors to this excess mortality.

Patients with schizophrenia not only have higher cancer-related mortality than the general population, but also, in general, higher disease-related mortality.^59–61^ These findings suggest that a comprehensive programme may be useful for the screening and treatment of diseases that individuals with schizophrenia are more likely to develop. Ideally, each region or country could consider designing a system in which information on individuals with schizophrenia is recorded in an integrated way. These individuals could undergo regular general check-ups and could also be examined with regard to common diseases. Each time they are assessed, their information could be recorded in the same integrated system. This could increase the likelihood of diagnosing diseases at early stages, and, with the data obtained over the years from people with schizophrenia, could also allow for the development of models for predicting the occurrence of disease. Such systems may exist in some countries, but they usually do not exist in low- and middle-income countries. However, if such integrated systems are not yet feasible, simpler and more immediately implementable strategies may still be useful. These may include educational packages for families of individuals with schizophrenia, containing information about healthy lifestyles, common physical health risks and the importance of cancer screening. Families could also be encouraged to support periodic screening and regular medical check-ups for affected individuals. Strengthening insurance systems to cover cancer screening and treatment services for individuals with schizophrenia may also help reduce delays in diagnosis and care. Future studies should examine the effectiveness of such practical interventions in different communities and healthcare settings.

Most of the available epidemiological data on schizophrenia are also from high-income countries.^62^ In our study, no studies from low- and middle-income countries were included. Based on our interpretation, this may partly reflect lower research funding and weaker registry-based research infrastructure in these countries. However, this finding should be interpreted cautiously, because other factors may also contribute, including publication of relevant studies in languages other than English, differences in data availability and limited access to linked psychiatric and mortality data.

The very high heterogeneity observed in several pooled analyses should be considered when interpreting these results. The pooled SMRs should not be understood as a single fixed estimate that applies equally across all countries, healthcare systems, time periods, cancer sites or patient groups. Rather, they provide an overall summary of the direction and approximate magnitude of excess cancer-related mortality in a heterogeneous body of evidence. Given the very high *I*^2^-values in several principal analyses, these pooled SMRs should be interpreted primarily as average summary measures rather than precise quantitative estimates directly applicable across settings. This heterogeneity may reflect differences in study design, population characteristics, methods of schizophrenia ascertainment, cancer-site composition, follow-up duration, cause-of-death registration and access to screening and cancer care. Therefore, the main clinical implication is not that one pooled estimate should be applied uniformly in all settings. Instead, the findings support considering individuals with schizophrenia as a high-risk population who may require more systematic physical health monitoring, earlier cancer detection and guideline-concordant cancer treatment. Where available, site-specific and gender-specific estimates may be more informative for clinical and public health planning than the overall pooled estimate.

Given the relatively small number of studies in most of our meta-analyses, conducting meta-regression is not particularly recommended from a methodological perspective, because statistical power is low and the risk of obtaining spurious associations (type 1 error) increases. Nevertheless, to obtain an overall and exploratory picture of the sources of heterogeneity, we performed meta-regression only in subgroups that included five or more, and from the outset we regarded these analyses as ‘non-definitive’ and lacking the power for causal inference. For this reason, the results of our meta-regressions are used more to provide a general view of the pattern of changes in effect sizes in relation to variables such as year of publication, sample size or follow-up duration, rather than as a basis for firm conclusions or clinical recommendations. Even in the limited number of cases where associations were statistically significant, these instances were few and their pattern did not recur across all groups; for example, year of publication might show an association with effect size in one cancer type or one gender group, whereas no such pattern is observed in other groups. This heterogeneity of patterns, together with the multiplicity of tests, highlights the possibility of the role of chance, type 1 error, random differences in study design and publication bias, and prevents us from interpreting these relationships as real and stable trends. Therefore, the meta-regression results in this study should be viewed with great caution and considered only as a basis for hypothesis generation and for guiding future research, rather than as a foundation for final conclusions.

The findings of this study are important because, with an integrated and comprehensive approach, they clarify the pattern of mortality from different cancers in patients with schizophrenia, both at an overall level and by cancer site. Simultaneously mapping the risk of death due to cancer in general and for specific cancers (such as cancers of the respiratory, gastrointestinal and genitourinary systems, etc.) may help inform the design of evidence-based programmes for screening and regular follow-up in this high-risk population. In other words, instead of relying on scattered reports about one or a few cancer types, the results of this study provide a coherent picture of the relative priority of each cancer group and may help inform screening priorities, allocating health system resources and developing clinical guidelines specifically for patients with schizophrenia.

### Strengths and limitations

In interpreting the results of this systematic review and meta-analysis, it is necessary to take into account both its strengths and its limitations. In terms of strengths, to the best of our knowledge, this is the first study that, with this level of breadth and detail, has systematically synthesised and estimated mortality from different types of cancer in patients with schizophrenia – both overall and by cancer site, and stratified by gender – and can therefore provide a relatively comprehensive picture to inform planning of screening programmes and preventive interventions in this population. In addition, our search strategy, which covered five major databases (PubMed, Scopus, Web of Science, PsycINFO and Embase) without any time restrictions, reduced the likelihood of missing important studies and increased the comprehensiveness of the review. Furthermore, we conducted a structured and relatively detailed quality assessment of all included studies with the NOS tool, and reported these results in full in the Supplementary Material, so that readers can properly take methodological quality into account when interpreting the findings.

At the same time, several important limitations should be noted. First, the number of studies was small in many subgroups, and from a methodological standpoint, meta-regression is not strongly recommended in such circumstances; therefore, we performed exploratory meta-regression only in groups that included five or more studies, and we explicitly regard these results as non-definitive and only as providing a ‘broad overview’ of potential sources of heterogeneity, rather than as a basis for firm conclusions. Second, in most studies the comparison group was the ‘general population’, which in principle can also include individuals with schizophrenia; this probably leads to a slight attenuation of the estimated excess risk (bias toward the null), although, given the low prevalence of schizophrenia in the general population, it is unlikely to have a major quantitative impact on the overall results. Third, because of practical constraints, we only included studies published in English. This may have introduced language bias and may also have contributed to the underrepresentation of evidence from non-English-speaking regions. Therefore, the findings should be interpreted with this restriction in mind. Fourth, in some of the earlier studies, the reported confidence intervals for SMRs were numerically unbalanced or not fully consistent with the corresponding point estimates. In these cases, after log-transformation and back-transformation, the confidence intervals generated by the statistical software were not always identical to the originally reported intervals. This occurred because some confidence intervals reported in the primary studies were numerically unbalanced or internally inconsistent with their point estimates. These cases were checked, and the differences were minor and did not materially affect the pooled estimates. This issue has also been noted in previous, similar meta-analysis^24^ and seems to reflect limitations in reporting in some of the older studies. Finally, the inherent heterogeneity in study designs, populations under study, time periods and methods of diagnosing schizophrenia and recording cause of death cannot be fully controlled, and although we used random-effects models and exploratory meta-regression to account for and explore this, the results of this study should be interpreted in light of these limitations rather than in isolation from them. In addition, a Grading of Recommendations Assessment, Development, and Evaluation assessment of the certainty of evidence for this systematic review was not performed.

## Supporting information

10.1192/bjo.2026.12068.sm001 Mahdavinoor et al. supplementary material 1 Mahdavinoor et al. supplementary material

10.1192/bjo.2026.12068.sm002 Mahdavinoor et al. supplementary material 2 Mahdavinoor et al. supplementary material

## Data Availability

The data supporting the findings of the study are available from the corresponding author, L.S., upon reasonable request.

## Supplementary material

The supplementary material is available online at https://doi.org/10.1192/bjo.2026.12068

## Table 1 Long description

A table comparing cancer-related mortality in individuals with schizophrenia by cancer type and gender. The table has 12 rows and 7 columns. Column headers are Cancer type, Number of studies, Pooled SMR, 95% CI, P-value, I^2 %, and P-value. Row labels include All, Male, Female, Breast, Respiratory, Gastrointestinal, Haematologic, Other, Skin and soft tissue, and Urogenital. Each row provides data for the respective cancer type and gender, including the number of studies, pooled standardised mortality ratio (SMR), 95% confidence interval (CI), P-value, and I^2 percentage. Notable trends include higher SMR values for respiratory and urogenital cancers in females and higher SMR values for skin and soft tissue cancers in both genders. The table shows varying levels of statistical significance across different cancer types and genders.

Navigate back to Table 1.

## Table 2 Long description

A table summarizing publication bias assessments for various cancer types using Begg’s and Egger’s tests. The table has 4 columns: Cancer type, n, Begg’s test, and Egger’s test. It includes data for different cancer types, gender-specific data, and specific cancer categories like breast, respiratory, gastrointestinal, haematologic, other, skin and soft tissue, and urogenital. Each row provides the number of studies (n) and the P-values for Begg’s and Egger’s tests. Notable entries include significant P-values for haematologic cancer in females for Egger’s test and various other cancer types with their respective P-values.

Navigate back to Table 2.

## Table 3 Long description

The table presents meta-regression analyses exploring potential sources of heterogeneity in cancer-related mortality. It includes coefficients, 95% confidence intervals, standard errors, and P-values for various variables across different cancer types and genders. The table has 36 rows and 5 columns. Column headers are Coefficient (β), 95% CI, s.e., and P-value. Row labels include categories such as All, Male, Female, Respiratory, Gastrointestinal, Haematologic, and Urogenital, with subcategories for gender-specific analyses. Each row provides data for the number of patients, year of publication, and follow-up duration. Notable trends include significant inverse associations for year of publication and follow-up duration in specific cancer types and genders, indicating potential sources of heterogeneity in standardized mortality ratios (SMRs).

Navigate back to Table 3.

## References

[1] Aker S , Kahve AC. What do psychiatrists think about renaming schizophrenia in Turkey? Schizophr Res 2022; 248: 8–13. 35907348 10.1016/j.schres.2022.07.009

[2] Ni L , Wu J , Long Y , Tao J , Xu J , Yuan X , et al. Mortality of site-specific cancer in patients with schizophrenia: a systematic review and meta-analysis. BMC Psychiatry 2019; 19: 323. 31660909 10.1186/s12888-019-2332-z PMC6816203

[3] Solmi M , Seitidis G , Mavridis D , Correll CU , Dragioti E , Guimond S , et al. Incidence, prevalence, and global burden of schizophrenia – data, with critical appraisal, from the Global Burden of Disease (GBD) 2019. Mol Psychiatry 2023; 28: 5319–27. 37500825 10.1038/s41380-023-02138-4

[4] Aker S , Böke Ö. , Oğuz G. Can renaming schizophrenia reduce negative attitudes toward patients in Turkey? Int J Soc Psychiatry 2016; 62: 311–5. 26719486 10.1177/0020764015621942

[5] Kadakia A , Catillon M , Fan Q , Williams G , Marden J , Anderson A , et al. The economic burden of schizophrenia in the United States. J Clin Psychiatry 2022; 83: 22m14458. 10.4088/JCP.22m14458 36244006

[6] Owen MJ , Sawa A , Mortensen PB. Schizophrenia. Lancet 2016; 388: 86–97. 26777917 10.1016/S0140-6736(15)01121-6 PMC4940219

[7] Orrico-Sánchez A , López-Lacort M , Muñoz-Quiles C , Sanfélix-Gimeno G , Díez-Domingo J. Epidemiology of schizophrenia and its management over 8-years period using real-world data in Spain. BMC Psychiatry 2020; 20: 149. 32248839 10.1186/s12888-020-02538-8 PMC7132863

[8] Hjorthøj C , Stürup AE , McGrath JJ , Nordentoft M. Years of potential life lost and life expectancy in schizophrenia: a systematic review and meta-analysis. Lancet Psychiatry 2017; 4: 295–301. 28237639 10.1016/S2215-0366(17)30078-0

[9] McNamee L , Mead G , MacGillivray S , Lawrie SM. Schizophrenia, poor physical health and physical activity: evidence-based interventions are required to reduce major health inequalities. Br J Psychiatry 2013; 203: 239–41. 24085733 10.1192/bjp.bp.112.125070

[10] Crump C , Winkleby MA , Sundquist K , Sundquist J. Comorbidities and mortality in persons with schizophrenia: a Swedish National Cohort Study. Am J Psychiatry 2013; 170: 324–33. 23318474 10.1176/appi.ajp.2012.12050599

[11] Pan Z , Zhou L , Chen Y , Su J , Duan X , Zhong S. Clinical factors for all-cause mortality in people with schizophrenia: a retrospective cohort study between 2013 and 2021. Asian J Psychiatry 2025; 104: 104357. 10.1016/j.ajp.2024.104357 39793478

[12] Dickerson F , Khan S , Origoni A , Rowe K , Katsafanas E , Harvin A , et al. Risk factors for natural cause mortality in schizophrenia. JAMA Netw Open 2024; 7: e2432401. 39254976 10.1001/jamanetworkopen.2024.32401 PMC11388031

[13] Mahdavinoor SMM , Kargar-Soleimanabad S , Bordbari AH , Mollaei A , Seddigh L , Sarihi S , et al. Investigating the relationship between schizophrenia and incidence risk of breast cancer: a systematic review and meta-analysis of cohort studies. Iran J Psychiatry 2024; 19: 441–52. 40589840 10.18502/ijps.v19i4.16559 PMC12206459

[14] Vancampfort D , Correll CU , Galling B , Probst M , De Hert M , Ward PB , et al. Diabetes mellitus in people with schizophrenia, bipolar disorder and major depressive disorder: a systematic review and large scale meta-analysis. World Psychiatry 2016; 15: 166–74. 27265707 10.1002/wps.20309 PMC4911762

[15] Smith DJ , Langan J , McLean G , Guthrie B , Mercer SW. Schizophrenia is associated with excess multiple physical-health comorbidities but low levels of recorded cardiovascular disease in primary care: cross-sectional study. BMJ Open 2013; 3: e002808. 10.1136/bmjopen-2013-002808 PMC3641427 23599376

[16] Xu D , Kong L , Zhang W , Hu L , Chen C , Li J , et al. Lower risk of liver cancer in patients with schizophrenia: a systematic review and meta-analysis of cohort studies. Oncotarget 2015; 8: 102328–35. 10.18632/oncotarget.21679 PMC5731958 29254248

[17] Raviv G , Laufer M , Baruch Y , Barak Y. Risk of prostate cancer in patients with schizophrenia. Compr Psychiatry 2014; 55: 1639–42. 24957959 10.1016/j.comppsych.2014.05.007

[18] Torrey EF. Prostate cancer and schizophrenia. Urology 2006; 68: 1280–3. 17141844 10.1016/j.urology.2006.08.1061

[19] Li H , Li J , Yu X , Zheng H , Sun X , Lu Y , et al. The incidence rate of cancer in patients with schizophrenia: a meta-analysis of cohort studies. Schizophr Res 2018; 195: 519–28. 28943096 10.1016/j.schres.2017.08.065

[20] Zhuo C , Zhuang H , Gao X , Triplett PT. Lung cancer incidence in patients with schizophrenia: meta-analysis. Br J Psychiatry 2019; 215: 704–11. 30806345 10.1192/bjp.2019.23 PMC7557637

[21] Wang Y , Wang L , Li X , Liu B , Zhao Q , Chen P , et al. Polymorphisms of XRCC4 are involved in reduced colorectal cancer risk in Chinese schizophrenia patients. BMC Cancer 2010; 10: 523. 20920336 10.1186/1471-2407-10-523 PMC2958939

[22] Ko YS , Tsai H-C , Chi MH , Su C-C , Lee IH , Chen PS , et al. Higher mortality and years of potential life lost of suicide in patients with schizophrenia. Psychiatry Res 2018; 270: 531–7. 30342411 10.1016/j.psychres.2018.09.038

[23] Perini G , Grigoletti L , Hanife B , Biggeri A , Tansella M , Amaddeo F. Cancer mortality among psychiatric patients treated in a community-based system of care: a 25-year case register study. Soc Psychiatry Psychiatr Epidemiol 2014; 49: 693–701. 24092521 10.1007/s00127-013-0765-0

[24] Zhuo C , Tao R , Jiang R , Lin X , Shao M. Cancer mortality in patients with schizophrenia: systematic review and meta-analysis. Br J Psychiatry 2017; 211: 7–13. 28596246 10.1192/bjp.bp.116.195776

[25] Higgins J , Thomas J , Chandler J , Cumpston M , Li T , Page M , et al. Cochrane Handbook for Systematic Reviews of Interventions. Wiley, 2019. 10.1002/14651858.ED000142 PMC10284251 31643080

[26] Tawfik GM , Dila KAS , Mohamed MYF , Tam DNH , Kien ND , Ahmed AM , et al. A step by step guide for conducting a systematic review and meta-analysis with simulation data. Trop Med Health 2019; 47: 46. 31388330 10.1186/s41182-019-0165-6 PMC6670166

[27] Page MJ , Moher D , Bossuyt PM , Boutron I , Hoffmann TC , Mulrow CD , et al. PRISMA 2020 explanation and elaboration: updated guidance and exemplars for reporting systematic reviews. BMJ 2021; 372: n160. 33781993 10.1136/bmj.n160 PMC8005925

[28] Stroup DF , Berlin JA , Morton SC , Olkin I , Williamson GD , Rennie D , et al. Meta-analysis of observational studies in epidemiology: a proposal for reporting. JAMA 2000; 283: 2008–12. 10789670 10.1001/jama.283.15.2008

[29] Wells G , Shea B , O’Connell D , Peterson J , Welch V , Losos M , et al. The Newcastle–Ottawa Scale (NOS) for Assessing the Quality of Nonrandomised Studies in Meta-analyses. Ottawa Hospital Research Institute, n.d. (https://www.ohri.ca/programs/clinical_epidemiology/oxford.asp).

[30] Breslow NE , Day NE. Statistical methods in cancer research. Volume II: the design and analysis of cohort studies. IARC Sci Publ 1987; 82: 1–406. 3329634

[31] Tran E , Rouillon F , Loze JY , Casadebaig F , Philippe A , Vitry F , et al. Cancer mortality in patients with schizophrenia: an 11-year prospective cohort study. Cancer 2009; 115: 3555–62. 19548261 10.1002/cncr.24383

[32] Kisely S , Crowe E , Lawrence D. Cancer-related mortality in people with mental illness. JAMA Psychiatry 2013; 70: 209–17. 23247556 10.1001/jamapsychiatry.2013.278

[33] Kredentser M , Martens P , Chochinov H , Prior H. Cause and rate of death in people with schizophrenia across the lifespan: a population-based study in Manitoba, Canada. J Clin Psychiatry 2014; 75: 154–61. 24602251 10.4088/JCP.13m08711

[34] Hiroeh U , Kapur N , Webb R , Dunn G , Mortensen PB , Appleby L. Deaths from natural causes in people with mental illness: a cohort study. J Psychosomat Res 2008; 64: 275–83. 10.1016/j.jpsychores.2007.09.008 18291242

[35] Castagnini A , Foldager L , Bertelsen A. Excess mortality of acute and transient psychotic disorders: comparison with bipolar affective disorder and schizophrenia. Acta Psychiatr Scand 2013; 128: 370–5. 23331302 10.1111/acps.12077

[36] Høye A , Jacobsen BK , Hansen V. Increasing mortality in schizophrenia: are women at particular risk? A follow-up of 1111 patients admitted during 1980–2006 in northern Norway. Schizophr Res 2011; 132: 228–32. 21868200 10.1016/j.schres.2011.07.021

[37] Heilä H , Haukka J , Suvisaari J , Lonnqvist J. Mortality among patients with schizophrenia and reduced psychiatric hospital care. Psychol Med 2005; 35: 725–32. 15918349 10.1017/s0033291704004118

[38] Mortensen PB , Juel K. Mortality and causes of death in first admitted schizophrenic patients. Br J Psychiatry 1993; 163: 183–9. 8075909 10.1192/bjp.163.2.183

[39] Ren J , Duan Y , Wang J , Sun Y , Wang M , Geng Z , et al. Mortality and excess life-years lostin patients with schizophrenia under community care: a 5-year follow-up cohort study. Braz J Psychiatry 2023; 45: 216–25. 36753614 10.47626/1516-4446-2022-2918 PMC10288468

[40] Saku M , Tokudome S , Ikeda M , Kono S , Makimoto K , Uchimura H , et al. Mortality in psychiatric patients, with a specific focus on cancer mortality associated with schizophrenia. Int J Epidemiol 1995; 24: 366–72. 7635598 10.1093/ije/24.2.366

[41] Drevinskaite M , Kaceniene A , Patasius A , Stukas R , Germanavicius A , Miseikyte E , et al. Cancer mortality and morbidity among patients with schizophrenia: a hospital-based cohort study, 1992–2020. Acta Psychiatr Scand 2024; 149: 234–43. 38173088 10.1111/acps.13651

[42] Olfson M , Gerhard T , Huang C , Crystal S , Stroup TS. Premature mortality among adults with schizophrenia in the United States. JAMA Psychiatry 2015; 72: 1172–81. 26509694 10.1001/jamapsychiatry.2015.1737

[43] Anderson C , Connelly J , Johnstone EC , Owens DGC. V. Cause of death. Br J Psychiatry 1991; 159: 30–3. 1840767

[44] Kisely S , Forsyth S , Lawrence D. Why do psychiatric patients have higher cancer mortality rates when cancer incidence is the same or lower? Austr N Z J Psychiatry 2015; 50: 254–63. 10.1177/0004867415577979 25829481

[45] Fors BM , Dag I , Kerstin B , Widerlöv B. Mortality among persons with schizophrenia in Sweden: an epidemiological study. Nord J Psychiatry 2007; 61: 252–9. 17763118 10.1080/08039480701414932

[46] Laursen T , Munk-Olsen T , Nordentoft M , Brøbech P. Increased mortality among patients admitted with major psychiatric disorders: a register-based study comparing mortality in unipolar depressive disorder, bipolar affective disorder, schizoaffective disorder, and schizophrenia. J Clin Psychiatry 2007; 68: 899–907. 17592915 10.4088/jcp.v68n0612

[47] Brown S , Kim M , Mitchell C , Inskip H. Twenty-five year mortality of a community cohort with schizophrenia. Br J Psychiatry 2010; 196: 116–21. 20118455 10.1192/bjp.bp.109.067512 PMC4560167

[48] Daumit GL , Anthony CB , Ford DE , Fahey M , Skinner EA , Lehman AF , et al. Pattern of mortality in a sample of Maryland residents with severe mental illness. Psychiatry Res 2010; 176: 242–5. 20207013 10.1016/j.psychres.2009.01.006 PMC2966471

[49] Lawrence D , Holman CD , Jablensky AV , Threfall TJ , Fuller SA. Excess cancer mortality in Western Australian psychiatric patients due to higher case fatality rates. Acta Psychiatr Scand 2000; 101: 382–8. 10823298 10.1034/j.1600-0447.2000.101005382.x

[50] Ösby U , Correia N , Brandt L , Ekbom A , Sparén P. Mortality and causes of death in schizophrenia in Stockholm County, Sweden. Schizophr Res 2000; 45: 21–8. 10978869 10.1016/s0920-9964(99)00191-7

[51] Zhong S , Pan Z , Su J , Duan X , Chen Y , Zhou L. Excess mortality in people with schizophrenia: 8-year population-based study in southern China. BJPsych Open 2025; 11: e251. 41128674 10.1192/bjo.2025.10866 PMC12569625

[52] Fang X , Yu H , Wu C , Tan J , Sun F , Qian N , et al. A retrospective and comprehensive analysis of excess life-years lost, mortality risk, and cause of death among severe mental illness in China. BMC Med 2025; 23: 571. 41121045 10.1186/s12916-025-04380-9 PMC12538965

[53] Worldometer. *Asia Population (2026)*. Worldometer, 2026 (https://www.worldometers.info/world-population/asia-population/).

[54] Davis LE , Bogner E , Coburn NG , Hanna TP , Kurdyak P , Groome PA , et al. Stage at diagnosis and survival in patients with cancer and a pre-existing mental illness: a meta-analysis. J Epidemiol Commun Health 2020; 74: 84. 10.1136/jech-2019-212311 31653661

[55] Irwin KE , Henderson DC , Knight HP , Pirl WF. Cancer care for individuals with schizophrenia. Cancer 2014; 120: 323–34. 24151022 10.1002/cncr.28431

[56] Irwin KE , Park ER , Shin JA , Fields LE , Jacobs JM , Greer JA , et al. Predictors of disruptions in breast cancer care for individuals with schizophrenia. Oncologist 2017; 22: 1374–82. 28559411 10.1634/theoncologist.2016-0489 PMC5679818

[57] Zhao H , Wu L , Yan G , Chen Y , Zhou M , Wu Y , et al. Inflammation and tumor progression: signaling pathways and targeted intervention. Signal Transd Target Ther 2021; 6: 263. 10.1038/s41392-021-00658-5 PMC8273155 34248142

[58] Momtazmanesh S , Zare-Shahabadi A , Rezaei N. Cytokine alterations in schizophrenia: an updated review. Front Psychiatry 2019; 10: 892. 31908647 10.3389/fpsyt.2019.00892 PMC6915198

[59] Pardamean E , Roan W , Iskandar KTA , Prayangga R , Hariyanto TI. Mortality from coronavirus disease 2019 (Covid-19) in patients with schizophrenia: a systematic review, meta-analysis and meta-regression. Gen Hosp Psychiatry 2022; 75: 61–7. 35182908 10.1016/j.genhosppsych.2022.01.010 PMC8813760

[60] Correll CU , Solmi M , Croatto G , Schneider LK , Rohani-Montez SC , Fairley L , et al. Mortality in people with schizophrenia: a systematic review and meta-analysis of relative risk and aggravating or attenuating factors. World Psychiatry 2022; 21: 248–71. 35524619 10.1002/wps.20994 PMC9077617

[61] Correll CU , Solmi M , Veronese N , Bortolato B , Rosson S , Santonastaso P , et al. Prevalence, incidence and mortality from cardiovascular disease in patients with pooled and specific severe mental illness: a large-scale meta-analysis of 3,211,768 patients and 113,383,368 controls. World Psychiatry 2017; 16: 163–80. 28498599 10.1002/wps.20420 PMC5428179

[62] Charlson FJ , Ferrari AJ , Santomauro DF , Diminic S , Stockings E , Scott JG , et al. Global epidemiology and burden of schizophrenia: findings from the global burden of disease study 2016. Schizophr Bull 2018; 44: 1195–203. 29762765 10.1093/schbul/sby058 PMC6192504

