# Supplementary material for: Cancer-related mortality among individuals with schizophrenia: systematic review and meta-analysis of cohort studies
Source: BJPsych Open. 2026 Aug 6;12(5):e206. doi: 10.1192/bjo.2026.12068 (PMC13458379; doi:10.1192/bjo.2026.12068)
Supplement: Supplementary file 1 [file S2056472426120687sup001.docx]

| **Domain and topic** | | | | | | | | | | | | |
| --- | --- | --- | --- | --- | --- | --- | --- | --- | --- | --- | --- | --- |
| Study quality | Total | **outcome** | | | **Comparability** | | **selection** | | | | Year | Author |
|  |  | **Adequacy of follow-up of cohorts** | **Was follow-up long enough for outcomes to occur?** | **Assessment of outcome** | **Comparability: additional factors** | **Comparability: age and sex** | **Outcome was not present at study start** | **Ascertainment of exposure** | **Selection of the non-exposed cohort** | **Representativeness of the exposed cohort** |  |  |
| Good | 8 | * | * | * |  | * | * | * | * | * | 2014 | Giovanni Perini |
| Good | 8 | * | * | * |  | * | * | * | * | * | 2009 | Eric Tran |
| Good | 8 | * | * | * |  | * | * | * | * | * | 2013 | Stephen Kisely |
| Good | 9 | * | * | * | * | * | * | * | * | * | 2014 | Maia S. Kredentser |
| Good | 8 | * | * | * |  | * | * | * | * | * | 2008 | Urara Hiroeh |
| Good | 8 | * | * | * |  | * | * | * | * | * | 2013 | A. Castagnini |
| Good | 8 | * | * | * |  | * | * | * | * | * | 2018 | Yu Shun Ko |
| Good | 8 | * | * | * |  | * | * | * | * | * | 2011 | Anne Høye |
| Good | 8 | * | * | * |  | * | * | * | * | * | 2005 | Hannele Heila |
| Good | 8 | * | * | * |  | * | * | * | * | * | 1993 | Preben Bo Mortensen |
| Fair | 6 | * |  | * |  | * | * | * |  | * | 2023 | Jintao Ren |
| Good | 7 | * | * | * |  | * | * | * |  | * | 1995 | Motohiro Saku, |
| Good | 8 | * | * | * |  | * | * | * | * | * | 2024 | M. Drevinskaite |
| Good | 8 | * |  | * | * | * | * | * | * | * | 2015 | Mark Olfson |
| Good | 8 | * | * | * |  | * | * | * | * | * | 1991 | C. Anderson |
| Good | 7 | * |  | * |  | * | * | * | * | * | 2015 | Steve Kisely |
| Good | 8 | * | * | * |  | * | * | * | * | * | 2007 | BJORN MILESSON FORS |
| Good | 8 | * | * | * |  | * | * | * | * | * | 2007 | Thomas Munk Laursen, |
| Good | 7 | * | * | * |  | * | * | * |  | * | 2010 | Steve Brown |
| Good | 7 | * |  | * |  | * | * | * | * | * | 2010 | Gail L. Daumit |
| Good | 8 | * | * | * |  | * | * | * | * | * | 2000 | D. Lawrence |
| Good | 8 | * | * | * |  | * | * | * | * | * | 2000 | Urban Osby |
| Good | 7 | * |  | * |  | * | * | * | * | * | 2025 | Zhong |
| Fair | 6 |  |  | * |  | * | * | * | * | * | 2025 | Xinyu Fang |
