# Supplementary material for: Cancer-related mortality among individuals with schizophrenia: systematic review and meta-analysis of cohort studies
Source: BJPsych Open. 2026 Aug 6;12(5):e206. doi: 10.1192/bjo.2026.12068 (PMC13458379; doi:10.1192/bjo.2026.12068)
Supplement: Supplementary file 2 [file S2056472426120687sup002.docx]

Table S1: Data Extraction File of Cancer-Related Mortality Among Individuals with Schizophrenia

| **Author (Year)** | **Country** | **Number of patients with schizophrenia (Male, Female)** | **Confirmation of Schizophrenia** | **Comparison population** | **Follow-up** | **Number of cancer deaths in patients with schizophrenia (Male, Female)** | **Confirmation of cancer deaths** | **Type of Cancer** | **Effect Size of both genders (95% CI)** | **Effect Size of men (95% CI)** | **Effect Size of female (95% CI)** |
| --- | --- | --- | --- | --- | --- | --- | --- | --- | --- | --- | --- |
| Giovanni Perini (2013) | Italy | 695 (NR, NR) | South Verona Psychiatric Case Register (PCR) | General Population | 1982–2006 | NR | municipal civil registries, the Mortality Registry of the Local Health District, and the Forensic Medicine | All cancer | SMR: 0.83 (0.5-1.3) | NR | NR |
| Giovanni Perini (2013) | Italy | 695 (NR, NR) | South Verona Psychiatric Case Register (PCR) | General Population | 1982–2006 | NR | municipal civil registries, the Mortality Registry of the Local Health District, and the Forensic Medicine | Oral cavity, lip. and pharynx | SMR: 0 | NR | NR |
| Giovanni Perini (2013) | Italy | 695 (NR, NR) | South Verona Psychiatric Case Register (PCR) | General Population | 1982–2006 | NR | municipal civil registries, the Mortality Registry of the Local Health District, and the Forensic Medicine | Stomach | SMR: 0 | NR | NR |
| Giovanni Perini (2013) | Italy | 695 (NR, NR) | South Verona Psychiatric Case Register (PCR) | General Population | 1982–2006 | NR | municipal civil registries, the Mortality Registry of the Local Health District, and the Forensic Medicine | Liver | SMR: 2.52 (0.7-6.73) | NR | NR |
| Giovanni Perini (2013) | Italy | 695 (NR, NR) | South Verona Psychiatric Case Register (PCR) | General Population | 1982–2006 | NR | municipal civil registries, the Mortality Registry of the Local Health District, and the Forensic Medicine | Pancreas | SMR: 1.19 (0.24-3.84) | NR | NR |
| Giovanni Perini (2013) | Italy | 695 (NR, NR) | South Verona Psychiatric Case Register (PCR) | General Population | 1982–2006 | NR | municipal civil registries, the Mortality Registry of the Local Health District, and the Forensic Medicine | Larynx | SMR: 0 | NR | NR |
| Giovanni Perini (2013) | Italy | 695 (NR, NR) | South Verona Psychiatric Case Register (PCR) | General Population | 1982–2006 | NR | municipal civil registries, the Mortality Registry of the Local Health District, and the Forensic Medicine | Pleura | SMR: 0 | NR | NR |
| Giovanni Perini (2013) | Italy | 695 (NR, NR) | South Verona Psychiatric Case Register (PCR) | General Population | 1982–2006 | NR | municipal civil registries, the Mortality Registry of the Local Health District, and the Forensic Medicine | Esophagus | SMR: 0 | NR | NR |
| Giovanni Perini (2013) | Italy | 695 (NR, NR) | South Verona Psychiatric Case Register (PCR) | General Population | 1982–2006 | NR | municipal civil registries, the Mortality Registry of the Local Health District, and the Forensic Medicine | Colon rectum | SMR: 0.53 (0.05-2.51) | NR | NR |
| Giovanni Perini (2013) | Italy | 695 (NR, NR) | South Verona Psychiatric Case Register (PCR) | General Population | 1982–2006 | NR | municipal civil registries, the Mortality Registry of the Local Health District, and the Forensic Medicine | Gallbladder and biliary tract | SMR: 0 | NR | NR |
| Giovanni Perini (2013) | Italy | 695 (NR, NR) | South Verona Psychiatric Case Register (PCR) | General Population | 1982–2006 | NR | municipal civil registries, the Mortality Registry of the Local Health District, and the Forensic Medicine | Other digestive system | SMR: 0 | NR | NR |
| Giovanni Perini (2013) | Italy | 695 (NR, NR) | South Verona Psychiatric Case Register (PCR) | General Population | 1982–2006 | NR | municipal civil registries, the Mortality Registry of the Local Health District, and the Forensic Medicine | Trachea, bronchus, lung | SMR: 0.24 (0.02-1.14) | NR | NR |
| Giovanni Perini (2013) | Italy | 695 (NR, NR) | South Verona Psychiatric Case Register (PCR) | General Population | 1982–2006 | NR | municipal civil registries, the Mortality Registry of the Local Health District, and the Forensic Medicine | Bones and connective tissue | SMR: 11.01(1.01-51.8) | NR | NR |
| Giovanni Perini (2013) | Italy | 695 (NR, NR) | South Verona Psychiatric Case Register (PCR) | General Population | 1982–2006 | NR | municipal civil registries, the Mortality Registry of the Local Health District, and the Forensic Medicine | Melanoma | SMR: 0 | NR | NR |
| Giovanni Perini (2013) | Italy | 695 (NR, NR) | South Verona Psychiatric Case Register (PCR) | General Population | 1982–2006 | NR | municipal civil registries, the Mortality Registry of the Local Health District, and the Forensic Medicine | Breast (woman) | SMR: 0.31 (0.03-1.47) | NR | NR |
| Giovanni Perini (2013) | Italy | 695 (NR, NR) | South Verona Psychiatric Case Register (PCR) | General Population | 1982–2006 | NR | municipal civil registries, the Mortality Registry of the Local Health District, and the Forensic Medicine | Prostate | SMR: 0.83 (0.5-1.3) | NR | NR |
| Giovanni Perini (2013) | Italy | 695 (NR, NR) | South Verona Psychiatric Case Register (PCR) | General Population | 1982–2006 | NR | municipal civil registries, the Mortality Registry of the Local Health District, and the Forensic Medicine | Bladder and kidney | SMR: 2.96 (0.6-9.57) | NR | NR |
| Giovanni Perini (2013) | Italy | 695 (NR, NR) | South Verona Psychiatric Case Register (PCR) | General Population | 1982–2006 | NR | municipal civil registries, the Mortality Registry of the Local Health District, and the Forensic Medicine | Brain and cranial nerves | SMR: 0 | NR | NR |
| Giovanni Perini (2013) | Italy | 695 (NR, NR) | South Verona Psychiatric Case Register (PCR) | General Population | 1982–2006 | NR | municipal civil registries, the Mortality Registry of the Local Health District, and the Forensic Medicine | Hodgkin lymphomas | SMR: 0 | NR | NR |
| Giovanni Perini (2013) | Italy | 695 (NR, NR) | South Verona Psychiatric Case Register (PCR) | General Population | 1982–2006 | NR | municipal civil registries, the Mortality Registry of the Local Health District, and the Forensic Medicine | Other skin | SMR: 0 (-0.01-17.6) | NR | NR |
| Giovanni Perini (2013) | Italy | 695 (NR, NR) | South Verona Psychiatric Case Register (PCR) | General Population | 1982–2006 | NR | municipal civil registries, the Mortality Registry of the Local Health District, and the Forensic Medicine | Uterus | SMR: 0 | NR | NR |
| Giovanni Perini (2013) | Italy | 695 (NR, NR) | South Verona Psychiatric Case Register (PCR) | General Population | 1982–2006 | NR | municipal civil registries, the Mortality Registry of the Local Health District, and the Forensic Medicine | Testis and other male genital system | SMR: NR | NR | NR |
| Giovanni Perini (2013) | Italy | 695 (NR, NR) | South Verona Psychiatric Case Register (PCR) | General Population | 1982–2006 | NR | municipal civil registries, the Mortality Registry of the Local Health District, and the Forensic Medicine | Non-Hodgkin lymphomas and leukemias | SMR: 11.72 (2.35-37.71) | NR | NR |
| Giovanni Perini (2013) | Italy | 695 (NR, NR) | South Verona Psychiatric Case Register (PCR) | General Population | 1982–2006 | NR | municipal civil registries, the Mortality Registry of the Local Health District, and the Forensic Medicine | Ill defined | SMR: 0 | NR | NR |
| Giovanni Perini (2013) | Italy | 695 (NR, NR) | South Verona Psychiatric Case Register (PCR) | General Population | 1982–2006 | NR | municipal civil registries, the Mortality Registry of the Local Health District, and the Forensic Medicine | Eye | SMR: NR | NR | NR |
| Eric Tran (2009) | France | 3434 (NR, NR) | Psychiatric Departments | General Population | 1993-2003 | 74 (46, 28) | National  Death Certificate | All Cancers | SMR: 1.5 (1.2-1.9) | SMR: 1.4 | SMR: 1.9 (1.4-2.8) |
| Eric Tran (2009) | France | 3434 (NR, NR) | Psychiatric Departments | General Population | 1993-2003 | 26 (23, 3) | National  Death Certificate | Lung | SMR: 2.1 (1.5-3.0) | SMR: 2.2 (1.6-3.3) | SMR: 1.5 |
| Eric Tran (2009) | France | 3434 (NR, NR) | Psychiatric Departments | General Population | 1993-2003 | 11 (NR, 11) | National  Death Certificate | Breast | SMR: 2.8 (1.6-4.9) | NR | SMR: 2.8 (1.6-4.9) |
| Eric Tran (2009) | France | 3434 (NR, NR) | Psychiatric Departments | General Population | 1993-2003 | 5 (1, 4) | National  Death Certificate | Colon | SMR: 2.2 | SMR: 0.7 | SMR: 5.0 (2.1-12.0) |
| Eric Tran (2009) | France | 3434 (NR, NR) | Psychiatric Departments | General Population | 1993-2003 | 4 (4, 0) | National  Death Certificate | Ear, nose, and throat | SMR: 4.4 (1.9-11.0) | SMR: 5.0 (2.1-12.0) | NR |
| Eric Tran (2009) | France | 3434 (NR, NR) | Psychiatric Departments | General Population | 1993-2003 | 3 (1, 2) | National  Death Certificate | Stomach | SMR: 2.3 | SMR: 1.0 | SMR: 6.7 (2.0-22.0) |
| Eric Tran (2009) | France | 3434 (NR, NR) | Psychiatric Departments | General Population | 1993-2003 | 3 (2, 1) | National  Death Certificate | Pancreas | SMR: 1.4 | SMR: 1.3 | SMR: 1.7 |
| Eric Tran (2009) | France | 3434 (NR, NR) | Psychiatric Departments | General Population | 1993-2003 | 3 (2, 1) | National  Death Certificate | Brain | SMR: 2.1 | SMR: 2.0 | SMR: 2.5 |
| Eric Tran (2009) | France | 3434 (NR, NR) | Psychiatric Departments | General Population | 1993-2003 | 2 (2, 0) | National  Death Certificate | Lymph | SMR: 1.7 | SMR: 2.5 | NR |
| Eric Tran (2009) | France | 3434 (NR, NR) | Psychiatric Departments | General Population | 1993-2003 | 2 (2, 0) | National  Death Certificate | Other digestive | SMR: 6.7 (2.0-22.0) | SMR: 10.0 (3.3-30.6) | NR |
| Eric Tran (2009) | France | 3434 (NR, NR) | Psychiatric Departments | General Population | 1993-2003 | 2 (2, 0) | National  Death Certificate | Bladder | SMR: 2.5 | SMR: 2.9 | NR |
| Eric Tran (2009) | France | 3434 (NR, NR) | Psychiatric Departments | General Population | 1993-2003 | 2 (NR, 2) | National  Death Certificate | Uterus | SMR: 2.5 | NR | 2.5 |
| Eric Tran (2009) | France | 3434 (NR, NR) | Psychiatric Departments | General Population | 1993-2003 | 2 (NR, 2) | National  Death Certificate | Ovary | SMR: 2.2 | NR | SMR: 2.2 |
| Eric Tran (2009) | France | 3434 (NR, NR) | Psychiatric Departments | General Population | 1993-2003 | 1 (1, NR) | National  Death Certificate | Testicle | SMR: 10.0 (2.1-49.0) | SMR: 10.0 (2.1-49.0) | NR |
| Eric Tran (2009) | France | 3434 (NR, NR) | Psychiatric Departments | General Population | 1993-2003 | 1 (1, 0) | National  Death Certificate | Blood | SMR: 0.6 | SMR: 1.0 | NR |
| Eric Tran (2009) | France | 3434 (NR, NR) | Psychiatric Departments | General Population | 1993-2003 | 1 (1, NR) | National  Death Certificate | Prostate | SMR: 1.4 | SMR: 1.4 | NR |
| Eric Tran (2009) | France | 3434 (NR, NR) | Psychiatric Departments | General Population | 1993-2003 | 1 (1, 0) | National  Death Certificate | Liver | SMR: 0.5 | SMR: 0.5 | NR |
| Eric Tran (2009) | France | 3434 (NR, NR) | Psychiatric Departments | General Population | 1993-2003 | 1 (0, 1) | National  Death Certificate | Skin | SMR: 1.7 | NR | SMR: 5.0 |
| Eric Tran (2009) | France | 3434 (NR, NR) | Psychiatric Departments | General Population | 1993-2003 | 4 (3, 1) | National  Death Certificate | Not determined | SMR: 0.5 | SMR: 0.5 | SMR: 0.5 |
| Stephen Kisely (2013) | Australia | 275 (129, 146) | The Hospital Morbidity Data System, Mental Health Information System, and WA Cancer Registry | General Population | 1998-2007 | 147 (72, 75) | Registrar General’s Death Registration Data | All Cancers | NR | RR: 2.21 (1.75-2.78) | RR: 1.77 (1.41-2.22) |
| Maia S. Kredentser (2014) | Canada | 9038 (4458, 4580) | Population Health Research Data Repository | General Population | 1999-2008 | NR | Population Health Research Data Repository | All Cancers | RR: 1.05 | NR | NR |
| Maia S. Kredentser (2014) | Canada | 9038 (4458, 4580) | Population Health Research Data Repository | General Population | 1999-2008 | NR | Population Health Research Data Repository | Lung | RR: 1.45 | NR | NR |
| Urara Hiroeh (2008) | Denmark | 2415 (1208, 1207) | Danish Psychiatric Central Register | General Population | 1973-1993 | 467 (181, 286) | National Causes of Death Register | All Cancers | NR | SMR: 106 (92-123) | SMR: 144 (128-162) |
| A. Castagnini (2013) | Denmark | 4576 (NR) | The Danish Psychiatric Central Register (DPCR) | General Population | 1995-2008 | 21 (NR) | Danish Register of Causes of Death | All Cancers | SMR: 1.5 (1.0-2.3) | NR | NR |
| Yu Shun Ko (2018) | Taiwan | 4298 (2297, 2001) | National Cheng Kung University Hospital in southern Taiwan | General Population | 1998-2010 | 60 (33, 27) | Taiwan Death Register (TDR) | Malignant neoplasms | SMR: 4.8 (3.5-5.9) | SMR: 4.8 (3.0-6.4) | SMR: 4.8 (2.9-6.6) |
| Hannele Heila (2005) | Finland | 58761 | NR | General Population | National Hospital Discharge Register | 2320 (1161, 1159) | "National Causes  of Death Register" | All Cancers | NR | RR: 1.50 (1.41-1.58) | RR: 1.48 (1.40-1.57) |
| Prebenbo Mortensen (1993) | Denmark | 9156 (5658, 3498) | Danish psychiatric hospital or department | General Population | 1970-1987 | 78 (27, 51) | Danish register of Causes of Death | All Cancers | NR | SMR: 0.81 (0.54-1.19) | SMR: 1.01 (0.75-1.33) |
| Jintao Ren (2023) | China | 80560 (40951, 39589) | National Community Care Program System | General Population | 2015-2019 | 1523 (NR) | National Cause of Death Registry Information System | All Cancers | SMR: 1.76 (0.75-2.78) | SMR: 2.19 (1.15-3.23) | SMR: 1.09 (0.81-1.97) |
| Motohiro Saku (1995) | Japan | 2268 (1433, 835) | Patients admitted to a National Mental Hospital | General Population | 1948-1985 | 41 (24, 17) | Koseki register is a Japanese family registration system | All Cancers | NR | SMR: 0.84 (0.54-1.25) | SMR: 1.37 (0.8-2.19) |
| M. Drevinskaite (2024) | Lithuania | 8553 (4319, 4234) | Vilnius Republican Psychiatric Hospital | General Population | 1992-2020 | 338 (151, 187) | Lithuanian Cancer Register | All Sites | NR | SMR: 0.82 (0.7-0.96) | SMR: 1.28 (1.11-1.48) |
| M. Drevinskaite (2024) | Lithuania | 8553 (4319, 4234) | Vilnius Republican Psychiatric Hospital | General Population | 1992-2020 | 1 (0, 1) | Lithuanian Cancer Register | Lip | NR | NR | SMR: 19.39 (2.73-137.68) |
| M. Drevinskaite (2024) | Lithuania | 8553 (4319, 4234) | Vilnius Republican Psychiatric Hospital | General Population | 1992-2020 | 4 (2, 2) | Lithuanian Cancer Register | Mouth and pharynx | NR | SMR: 0.16 (0.04-0.64) | SMR: 1.07 (0.27-4.27) |
| M. Drevinskaite (2024) | Lithuania | 8553 (4319, 4234) | Vilnius Republican Psychiatric Hospital | General Population | 1992-2020 | 3 (1, 2) | Lithuanian Cancer Register | Oesophagus | NR | SMR: 0.13 (0.02-0.9) | SMR: 1.81 (0.45-7.22) |
| M. Drevinskaite (2024) | Lithuania | 8553 (4319, 4234) | Vilnius Republican Psychiatric Hospital | General Population | 1992-2020 | 24 (14, 10) | Lithuanian Cancer Register | Stomach | NR | SMR: 0.8 (0.47-1.35) | SMR: 0.91 (0.49-1.68) |
| M. Drevinskaite (2024) | Lithuania | 8553 (4319, 4234) | Vilnius Republican Psychiatric Hospital | General Population | 1992-2020 | 3 (1, 2) | Lithuanian Cancer Register | Other digestive organs | NR | SMR: 0.93 (0.13-6.62) | SMR: 1.63 (0.41-6.51) |
| M. Drevinskaite (2024) | Lithuania | 8553 (4319, 4234) | Vilnius Republican Psychiatric Hospital | General Population | 1992-2020 | 11 (3, 8) | Lithuanian Cancer Register | Colon | NR | SMR: 0.37 (0.12-1.15) | SMR: 0.88 (0.44-1.77) |
| M. Drevinskaite (2024) | Lithuania | 8553 (4319, 4234) | Vilnius Republican Psychiatric Hospital | General Population | 1992-2020 | 15 (9, 6) | Lithuanian Cancer Register | Rectum, rectosigmoid, anus | NR | SMR: 1.11 (0.58-2.13) | SMR: 0.85 (0.38-1.9) |
| M. Drevinskaite (2024) | Lithuania | 8553 (4319, 4234) | Vilnius Republican Psychiatric Hospital | General Population | 1992-2020 | 15 (12, 3) | Lithuanian Cancer Register | Liver | NR | SMR: 2.49 (1.41-4.38) | SMR: 1.1 (0.36-3.43) |
| M. Drevinskaite (2024) | Lithuania | 8553 (4319, 4234) | Vilnius Republican Psychiatric Hospital | General Population | 1992-2020 | 6 (2, 4) | Lithuanian Cancer Register | Gallbladder, bile ducts | NR | SMR: 1.78 (0.44-7.11) | SMR: 1.76 (0.66-4.68) |
| M. Drevinskaite (2024) | Lithuania | 8553 (4319, 4234) | Vilnius Republican Psychiatric Hospital | General Population | 1992-2020 | 9 (3, 6) | Lithuanian Cancer Register | Pancreas | NR | SMR: 0.3 (0.1-0.92) | SMR: 0.71 (0.32-1.59) |
| M. Drevinskaite (2024) | Lithuania | 8553 (4319, 4234) | Vilnius Republican Psychiatric Hospital | General Population | 1992-2020 | 0 | Lithuanian Cancer Register | Nasal cavity, middle ear, accessory sinuses | NR | NR | NR |
| M. Drevinskaite (2024) | Lithuania | 8553 (4319, 4234) | Vilnius Republican Psychiatric Hospital | General Population | 1992-2020 | 2 (2, 0) | Lithuanian Cancer Register | Larynx | NR | SMR: 0.32 (0.08-1.29) | NR |
| M. Drevinskaite (2024) | Lithuania | 8553 (4319, 4234) | Vilnius Republican Psychiatric Hospital | General Population | 1992-2020 | 55 (42, 13) | Lithuanian Cancer Register | Lung, trachea | NR | SMR: 0.85 (0.63-1.15) | SMR: 1.34 (0.78-2.3) |
| M. Drevinskaite (2024) | Lithuania | 8553 (4319, 4234) | Vilnius Republican Psychiatric Hospital | General Population | 1992-2020 | 1 (1, 0) | Lithuanian Cancer Register | Bone and connective tissue | NR | SMR: 0.55 (0.08-3.91) | NR |
| M. Drevinskaite (2024) | Lithuania | 8553 (4319, 4234) | Vilnius Republican Psychiatric Hospital | General Population | 1992-2020 | 7 (5, 2) | Lithuanian Cancer Register | Skin, melanoma | NR | SMR: 2.32 (0.97-5.58) | SMR: 0.88 (0.22-3.5) |
| M. Drevinskaite (2024) | Lithuania | 8553 (4319, 4234) | Vilnius Republican Psychiatric Hospital | General Population | 1992-2020 | 1 (0, 1) | Lithuanian Cancer Register | Skin, non-melanoma | NR | NR | SMR: 1.69 (0.24-11.99) |
| M. Drevinskaite (2024) | Lithuania | 8553 (4319, 4234) | Vilnius Republican Psychiatric Hospital | General Population | 1992-2020 | 50 (0, 50) | Lithuanian Cancer Register | Breast | NR | NR | SMR: 1.9 (1.44-2.51) |
| M. Drevinskaite (2024) | Lithuania | 8553 (4319, 4234) | Vilnius Republican Psychiatric Hospital | General Population | 1992-2020 | 1 (NR, 1) | Lithuanian Cancer Register | Vulva | NR | NR | SMR: 1.19 (0.17-8.43) |
| M. Drevinskaite (2024) | Lithuania | 8553 (4319, 4234) | Vilnius Republican Psychiatric Hospital | General Population | 1992-2020 | 10 (NR, 10) | Lithuanian Cancer Register | Cervix uteri | NR | NR | SMR: 0.92 (0.49-1.71) |
| M. Drevinskaite (2024) | Lithuania | 8553 (4319, 4234) | Vilnius Republican Psychiatric Hospital | General Population | 1992-2020 | 11 (NR, 11) | Lithuanian Cancer Register | Corpus uteri | NR | NR | SMR: 1.75 (0.97-3.16) |
| M. Drevinskaite (2024) | Lithuania | 8553 (4319, 4234) | Vilnius Republican Psychiatric Hospital | General Population | 1992-2020 | 15 (NR, 15) | Lithuanian Cancer Register | Ovary | NR | NR | SMR: 1.2 (0.72-1.99) |
| M. Drevinskaite (2024) | Lithuania | 8553 (4319, 4234) | Vilnius Republican Psychiatric Hospital | General Population | 1992-2020 | 12 (12, NR) | Lithuanian Cancer Register | Prostate | NR | SMR: 0.89 (0.51-1.57) | NR |
| M. Drevinskaite (2024) | Lithuania | 8553 (4319, 4234) | Vilnius Republican Psychiatric Hospital | General Population | 1992-2020 | 2 (2, NR) | Lithuanian Cancer Register | Testis | NR | SMR: 4.59 (1.15-18.35) | NR |
| M. Drevinskaite (2024) | Lithuania | 8553 (4319, 4234) | Vilnius Republican Psychiatric Hospital | General Population | 1992-2020 | 0 (0, 0) | Lithuanian Cancer Register | Other female and male genital organs | NR | NR | NR |
| M. Drevinskaite (2024) | Lithuania | 8553 (4319, 4234) | Vilnius Republican Psychiatric Hospital | General Population | 1992-2020 | 9 (4, 5) | Lithuanian Cancer Register | Kidney | NR | SMR: 0.54 (0.2-1.45) | SMR: 1.28 (0.53-3.07) |
| M. Drevinskaite (2024) | Lithuania | 8553 (4319, 4234) | Vilnius Republican Psychiatric Hospital | General Population | 1992-2020 | 1 (1, 0) | Lithuanian Cancer Register | Other urinary tract | NR | SMR: 2.46 (0.35-17.45) | NR |
| M. Drevinskaite (2024) | Lithuania | 8553 (4319, 4234) | Vilnius Republican Psychiatric Hospital | General Population | 1992-2020 | 6 (5, 1) | Lithuanian Cancer Register | Urinary bladder | NR | SMR: 0.91 (0.38-2.19) | SMR: 0.67 (0.09-4.74) |
| M. Drevinskaite (2024) | Lithuania | 8553 (4319, 4234) | Vilnius Republican Psychiatric Hospital | General Population | 1992-2020 | 0 (0, 0) | Lithuanian Cancer Register | Eye and adnexa | NR | NR | NR |
| M. Drevinskaite (2024) | Lithuania | 8553 (4319, 4234) | Vilnius Republican Psychiatric Hospital | General Population | 1992-2020 | 14 (7, 7) | Lithuanian Cancer Register | Central nervous system | NR | SMR: 1.2 (0.57-2.52) | SMR: 1.24 (0.59-2.6) |
| M. Drevinskaite (2024) | Lithuania | 8553 (4319, 4234) | Vilnius Republican Psychiatric Hospital | General Population | 1992-2020 | 0 (0, 0) | Lithuanian Cancer Register | Thyroid | NR | NR | NR |
| M. Drevinskaite (2024) | Lithuania | 8553 (4319, 4234) | Vilnius Republican Psychiatric Hospital | General Population | 1992-2020 | 0 (0, 0) | Lithuanian Cancer Register | Other endocrine organs | NR | NR | NR |
| M. Drevinskaite (2024) | Lithuania | 8553 (4319, 4234) | Vilnius Republican Psychiatric Hospital | General Population | 1992-2020 | 27 (13, 14) | Lithuanian Cancer Register | Other and ill-defined sites | NR | SMR: 1.77 (1.03-3.04) | SMR: 2.57 (1.52-4.34) |
| M. Drevinskaite (2024) | Lithuania | 8553 (4319, 4234) | Vilnius Republican Psychiatric Hospital | General Population | 1992-2020 | 3 (1, 2) | Lithuanian Cancer Register | Non-Hodgkin lymphoma | NR | SMR: 1.78 (0.25-12.61) | SMR: 5.05 (1.26-20.18) |
| M. Drevinskaite (2024) | Lithuania | 8553 (4319, 4234) | Vilnius Republican Psychiatric Hospital | General Population | 1992-2020 | 8 (3, 5) | Lithuanian Cancer Register | Hodgkin lymphoma | NR | SMR: 1.08 (0.35-3.34) | SMR: 1.95 (0.81-4.68) |
| M. Drevinskaite (2024) | Lithuania | 8553 (4319, 4234) | Vilnius Republican Psychiatric Hospital | General Population | 1992-2020 | 0 (0, 0) | Lithuanian Cancer Register | Other and unspecified malignant neoplasms of lymphoid, haematopoietic and related tissue | NR | NR | NR |
| M. Drevinskaite (2024) | Lithuania | 8553 (4319, 4234) | Vilnius Republican Psychiatric Hospital | General Population | 1992-2020 | 4 (2, 2) | Lithuanian Cancer Register | Myeloma | NR | SMR: 1.14 (0.29-4.56) | SMR: 0.84 (0.21-3.37) |
| M. Drevinskaite (2024) | Lithuania | 8553 (4319, 4234) | Vilnius Republican Psychiatric Hospital | General Population | 1992-2020 | 8 (4, 4) | Lithuanian Cancer Register | Leukaemia | NR | SMR: 0.82 (0.31-2.18) | SMR: 0.81 (0.3-2.15) |
| Mark Olfson (2015) | USA | 1138853 (610711, 528097) | National Medicaid  Analytic eXtract (MAX) data | General Population | 2001-2007 | 9638 (4767, 4871) | National Death Index | All Cancers | SMR: 1.8 (1.7-1.8) | SMR: 1.7 (1.7-1.8) | SMR: 1.8 (1.8-1.8) |
| Mark Olfson (2015) | USA | 1138853 (610711, 528097) | National Medicaid  Analytic eXtract (MAX) data | General Population | 2001-2007 | 3595 (2021, 1574) | National Death Index | Lung | SMR: 2.4 (2.4-2.5) | SMR: 2.4 (2.3-2.5) | SMR: 2.5 (2.4-2.6) |
| Mark Olfson (2015) | USA | 1138853 (610711, 528097) | National Medicaid  Analytic eXtract (MAX) data | General Population | 2001-2007 | 679 (338, 341) | National Death Index | Colon | SMR: 1.7 (1.6-1.8) | SMR: 1.6 (1.4-1.8) | SMR: 1.9 (1.7-2.1) |
| Mark Olfson (2015) | USA | 1138853 (610711, 528097) | National Medicaid  Analytic eXtract (MAX) data | General Population | 2001-2007 | 995 (16, 979) | National Death Index | Breast | SMR: 1.6 (1.5-1.7) | SMR: 3.6 (1.8-5.4) | SMR: 1.6 (1.5-1.7) |
| Mark Olfson (2015) | USA | 1138853 (610711, 528097) | National Medicaid  Analytic eXtract (MAX) data | General Population | 2001-2007 | 315 (230, 85) | National Death Index | Liver | SMR: 1.4 (1.2-1.5) | SMR: 1.3 (1.1-1.5) | SMR: 1.5 (1.2-1.9) |
| Mark Olfson (2015) | USA | 1138853 (610711, 528097) | National Medicaid  Analytic eXtract (MAX) data | General Population | 2001-2007 | 401 (227, 174) | National Death Index | Pancreas | SMR: 1.4 (1.2-1.5) | SMR: 1.3 (1.2-1.5) | SMR: 1.4 (1.2-1.6) |
| Mark Olfson (2015) | USA | 1138853 (610711, 528097) | National Medicaid  Analytic eXtract (MAX) data | General Population | 2001-2007 | 648 (371, 277) | National Death Index | Hematologic | SMR: 1.4 (1.3-1.6) | SMR: 1.4 (1.3-1.6) | SMR: 1.5 (1.3-1.7) |
| Mark Olfson (2015) | USA | 1138853 (610711, 528097) | National Medicaid  Analytic eXtract (MAX) data | General Population | 2001-2007 | 3005 (1564, 1441) | National Death Index | Other cancers | SMR: 1.5 (1.4-1.5) | SMR: 1.4 (1.3-1.7) | SMR: 1.6 (1.5-1.7) |
| C. Anderson (1991) | UK | 532 (291, 241) | In-patient or day-patient care in the Psychiatric Unit, Northwick Park Hospital, or from wards in Shenley Hospital, which admitted patients from Harrow | General Population | 1981-1990 | 4 (2, 2) | Obtained from death certificates, medical case notes, and coroner's reports | All Cancers | NR | SMR: 0.62 (0.07-2.33) | SMR: 0.46 (0.05-1.65) |
| Steve Kisely (2015) | Australia | NR | Queensland Hospital Admitted Patients’ Data Collection, Queensland Client Event Services Application | General Population | 2002-2007 | 87 (NR, NR) | Queensland Cancer Registry,Registrar General’s Death Registration Data | All Cancers | RR: 1.43 (1.1-1.75) | NR | NR |
| Bjorn Milesson Fors (2007) | Sweden | 255 (146, 109) | Hospital admission registers and records from outpatient care facilities, general practitioners, retirement homes, social agencies, prisons and district nurses were used | General Population | 1991-2000 | NR | Swedish Causes of Death register | All Cancers | RR: 1.2 | RR: 1.1 | RR: 1.3 |
| Thomas Munk Laursen (2007) | Denmark | 17660 (NR, NR) | The Danish Psychiatric Central Register | General Population | 1973-2001 | NR | The Danish Cause of Death register | All Cancers | NR | MRR: 1.24 (1.08-1.43) | MRR: 1.32 (1.18-1.48) |
| Steve Brown (2010) | UK | 370 (213, 157) | National Health Service (NHS) psychiatric services | General Population | 1981-2006 | 30 (20, 10) | Hampshire Partnership NHS Trust case notes, general practitioner or mental health worker | Neoplasms | SMR: 1.49 (1.00-2.12) | SMR: 1.93 (1.18-2.98) | SMR: 1.02 (0.49-1.88) |
| Steve Brown (2010) | UK | 370 (213, 157) | National Health Service (NHS) psychiatric services | General Population | 1981-2006 | 13 (NR, NR) | Hampshire Partnership NHS Trust case notes, general practitioner or mental health worker | Lung | SMR: 2.65 (1.41-4.53) | NR | NR |
| Steve Brown (2010) | UK | 370 (213, 157) | National Health Service (NHS) psychiatric services | General Population | 1981-2006 | 4 (NR, 4) | Hampshire Partnership NHS Trust case notes, general practitioner or mental health worker | Breast | SMR: 1.96 (0.54-5.03) | NR | NR |
| Gail L. Daumit (2010) | USA | NR | Medicaid administrative claims data | General Population | 1994-2001 | NR | National Death Index | Malignant Neoplasms | SMR: 1.4 (1.3-1.5) | NR | NR |
| D. Lawrence (2001) | Western Australia | NR | Western Australian Mental Health Information System (MHIS) | General Population | 1982-1995 | NR | Western Australian Cancer Register, the Western Australian Linked Database | All Cancers | NR | RR: 0.9 (0.71-1.8) | RR: 1.19 (1.05-1.4) |
| Urban O¨sby (2000) | Sweden | 7784 (3929, 3855) | Swedish psychiatric in-patient register | General Population | 1973-1995 | 225 (79, 146) | National cause-of-death register | All Cancers | NR | SMR: 1.1 (0.8-1.3) | SMR: 1.3 (1.1-1.5) |
| Urban O¨sby (2000) | Sweden | 7784 (3929, 3855) | Swedish psychiatric in-patient register | General Population | 1973-1995 | NR | National cause-of-death register | Prostate | NR | SMR: 0.86 (0.4-1.7) | NR |
| Urban O¨sby (2000) | Sweden | 7784 (3929, 3855) | Swedish psychiatric in-patient register | General Population | 1973-1995 | NR | National cause-of-death register | Lung | NR | SMR: 0.73 (0.4-1.3) | NR |
| Urban O¨sby (2000) | Sweden | 7784 (3929, 3855) | Swedish psychiatric in-patient register | General Population | 1973-1995 | NR | National cause-of-death register | Breast | NR | NR | SMR: 1.6 (1.1-2.3) |
| Urban O¨sby (2000) | Sweden | 7784 (3929, 3855) | Swedish psychiatric in-patient register | General Population | 1973-1995 | NR | National cause-of-death register | Urogenital | NR | NR | SMR: 1.5 (1.1-2.2) |
| Urban O¨sby (2000) | Sweden | 7784 (3929, 3855) | Swedish psychiatric in-patient register | General Population | 1973-1995 | NR | National cause-of-death register | cervix | NR | NR | SMR: 3.3 (0.8-4.3) |
| Urban O¨sby (2000) | Sweden | 7784 (3929, 3855) | Swedish psychiatric in-patient register | General Population | 1973-1995 | NR | National cause-of-death register | corpus uteri | NR | NR | SMR: 2.5 (0.7-4.8) |
| Xinyu Fang (2025) | China | 251976 (113465, 138511) | Jiangsu Province Management Information System | General Population | 2014-2022 | 4316 | Cause-of-death data obtained from the national Mortality Registration System of the China Center for Disease Control and Prevention (CDC) | Neoplasm | SMR: 1.14 (1.11-1.18) | NR | NR |
| Shaoling Zhong (2025) | China | 32600 (16764, 15836) | National Information System for Psychosis (NISP) | General Population | 2014-2021 | 549 (316, 233) | Causes of death from the national mortality registry (Guangzhou CDC) | Neoplasm | SMR: 1.14 (1.04-1.24) | SMR: 1.13 (1.01-1.27) | SMR: 1.14 (1.00-1.29) |

Figure S1: Forest plot of all-cancer–related mortality

Figure S2: Forest plot of all-cancer–related mortality in males

Figure S3: Forest plot of all-cancer–related mortality in females.

Figure S4: Forest plot of breast cancer–related mortality.

Figure S5: Forest plot of breast cancer–related mortality in females.

Figure S6: Forest plot of respiratory cancer–related mortality.

Figure S7: Forest plot of respiratory cancer–related mortality in males.

Figure S8: Forest plot of respiratory cancer–related mortality in females.

Figure S9: Forest plot of gastrointestinal cancer–related mortality.

Figure S10: Forest plot of gastrointestinal cancer–related mortality in males.

Figure S11: Forest plot of gastrointestinal cancer–related mortality in females.

Figure S12: Forest plot of hematological cancer–related mortality.

Figure S13: Forest plot of hematological cancer–related mortality in males.

Figure S14: Forest plot of hematological cancer–related mortality in females.

Figure S15: Forest plot of other cancer–related mortality.

Figure S16: forest plot for the other cancer based on male.

Figure S17: Forest plot of other cancer–related mortality in females.

Figure S18: Forest plot of skin and soft tissue cancer–related mortality.

Figure S19: Forest plot of skin and soft tissue cancer–related mortality in males.

Figure S20: Forest plot of skin and soft tissue cancer–related mortality in females

Figure S21: Forest plot of urogenital cancer–related mortality.

Figure S22: Forest plot of urogenital cancer–related mortality in males.

Figure S23: forest plot for the Urogenital cancer based on female.

Figure S24: Funnel plot of all-cancer–related mortality

Figure S25: Funnel plot of all-cancer–related mortality in males

Figure S26: Funnel plot of all-cancer–related mortality in females

Figure S27: Funnel plot of breast cancer–related mortality

Figure S28: Funnel plot of breast cancer–related mortality in females

Figure S29: Funnel plot of respiratory cancer–related mortality

Figure S30: Funnel plot of respiratory cancer–related mortality in males

Figure S31: Funnel plot of respiratory cancer–related mortality in females

Figure S32: Funnel plot of gastrointestinal cancer–related mortality

Figure S33: Funnel plot of gastrointestinal cancer–related mortality in males

Figure S34: Funnel plot of gastrointestinal cancer–related mortality in females

Figure S35: Funnel plot of hematological cancer–related mortality

Figure S36: Funnel plot of hematological cancer–related mortality in males

Figure S37: Funnel plot of hematological cancer–related mortality in females

Figure S38: Funnel plot of skin and soft tissue cancer–related mortality

Figure S39: Funnel plot of skin and soft tissue cancer–related mortality in males

Figure S40: Funnel plot of skin and soft tissue cancer–related mortality in females

Figure S41: Funnel plot of urogenital cancer–related mortality

Figure S42: Funnel plot of urogenital cancer–related mortality in males

Figure S43: Funnel plot of urogenital cancer–related mortality in females

Figure S44: Funnel plot of other cancer–related mortality

Figure S45: Funnel plot of other cancer–related mortality in males

Figure S46: Funnel plot of other cancer–related mortality in females

Table S2: Full Search Strategy and Results

| **Database** | **Date searched** | **Syntax** | **Initial (N)** | **Update (N)** | **Total (N)** |
| --- | --- | --- | --- | --- | --- |
| PubMed | Nov 2024/ Dec 2025 | [(Mortality[MeSH Terms] OR Death[Title/Abstract] OR Mortality[Title/Abstract]) AND (Cancer[MeSH Terms] OR Cancer*[Title/Abstract] OR Neoplasm*[Title/Abstract] OR Tumor*[Title/Abstract] OR Malignancy*[Title/Abstract] OR Carcinoma*[Title/Abstract] OR Gland*[Title/Abstract]) AND ("Dementia Praecox"[Title/Abstract] OR "Schizophre*"[Title/Abstract] OR psychosis[Title/Abstract])] | 475 | 48 | 523 |
| Scopus | Nov 2024/ Dec 2025 | ( TITLE-ABS-KEY ( Death ) OR TITLE-ABS-KEY ( Mortality ) AND TITLE-ABS-KEY ( cancer* ) OR TITLE-ABS-KEY ( neoplasm* ) OR TITLE-ABS-KEY ( tumor* ) OR TITLE-ABS-KEY ( malignancy* ) OR TITLE-ABS-KEY ( carcinoma* ) OR TITLE-ABS-KEY ( gland* ) AND TITLE-ABS-KEY ( "Dementia Praecox" ) OR TITLE-ABS-KEY ( "Schizophre*" ) OR TITLE-ABS-KEY ( psychosis ) ) | 1954 | 164 | 2118 |
| Web of Science | Nov 2024/ Dec 2025 | #7: ALL=("Dementia Praecox" ) OR ALL=(Schizophre*) OR ALL=(psychosis)  #8: ALL=(Mortality) OR ALL=(Death)  #9: ALL=(Cancer*) OR ALL=(Neoplasm*) OR ALL=(Tumor*) OR ALL=(Malignancy*) OR ALL=(Carcinoma*) OR ALL=(Gland*)  #7 and #8 and #9 | 941 | 161 | 1102 |
| Embase | Nov 2024/ Dec 2025 | (Death:ti,ab,kw OR Mortality:ti,ab,kw) AND ('dementia praecox':ti,ab,kw OR 'schizophre*':ti,ab,kw OR psychosis:ti,ab,kw) AND (cancer*:ti,ab,kw OR neoplasm*:ti,ab,kw OR tumor*:ti,ab,kw OR malignancy*:ti,ab,kw OR carcinoma*:ti,ab,kw OR gland*:ti,ab,kw) | 739 | 118 | 857 |
| PsycINFO | Nov 2024/ Dec 2025 | (TX Cancer* OR TX Neoplasm* OR TX Tumor* OR TX Malignancy* OR TX Carcinoma* OR TX Gland*) AND (TX “Dementia Praecox” OR TX Schizophre* OR TX psychosis) AND (TX Mortality OR TX Death) | 330 | 23 | 353 |
